# Impact of flavivirus vaccine-induced immunity on primary Zika virus antibody response in humans

**DOI:** 10.1371/journal.pntd.0008034

**Published:** 2020-02-04

**Authors:** Stefan Malafa, Iris Medits, Judith H. Aberle, Stephan W. Aberle, Denise Haslwanter, Georgios Tsouchnikas, Silke Wölfel, Kristina L. Huber, Elena Percivalle, Pascal Cherpillod, Melissa Thaler, Lena Roßbacher, Michael Kundi, Franz X. Heinz, Karin Stiasny

**Affiliations:** 1 Center for Virology, Medical University of Vienna, Vienna, Austria; 2 Bundeswehr Institute of Microbiology, Munich, Germany; Center of Infection Research (DZIF) Partner, Munich, Germany; 3 Division of Infectious Diseases and Tropical Medicine, Ludwig Maximilian University (LMU), Munich, Germany; 4 Molecular Virology Unit, Microbiology and Virology Department, Fondazione IRCCS Policlinico San Matteo, Pavia, Italy; 5 Laboratory of Virology, Laboratory Medicine Division, Geneva University Hospitals, Geneva, Switzerland; 6 Center for Public Health, Medical University of Vienna, Vienna, Austria; Duke-NUS GMS, SINGAPORE

## Abstract

**Background:**

Zika virus has recently spread to South- and Central America, causing congenital birth defects and neurological complications. Many people at risk are flavivirus pre-immune due to prior infections with other flaviviruses (e.g. dengue virus) or flavivirus vaccinations. Since pre-existing cross-reactive immunity can potentially modulate antibody responses to Zika virus infection and may affect the outcome of disease, we analyzed fine-specificity as well as virus-neutralizing and infection-enhancing activities of antibodies induced by a primary Zika virus infection in flavivirus-naïve as well as yellow fever- and/or tick-borne encephalitis-vaccinated individuals.

**Methodology:**

Antibodies in sera from convalescent Zika patients with and without vaccine-induced immunity were assessed by ELISA with respect to Zika virus-specificity and flavivirus cross-reactivity. Functional analyses included virus neutralization and infection-enhancement. The contribution of IgM and cross-reactive antibodies to these properties was determined by depletion experiments.

**Principal findings:**

Pre-existing flavivirus immunity had a strong influence on the antibody response in primary Zika virus infections, resulting in higher titers of broadly flavivirus cross-reactive antibodies and slightly lower levels of Zika virus-specific IgM. Antibody-dependent enhancement (ADE) of Zika virus was mediated by sub-neutralizing concentrations of specific IgG but not by cross-reactive antibodies. This effect was potently counteracted by the presence of neutralizing IgM. Broadly cross-reactive antibodies were able to both neutralize and enhance infection of dengue virus but not Zika virus, indicating a different exposure of conserved sequence elements in the two viruses.

**Conclusions:**

Our data point to an important role of flavivirus-specific IgM during the transient early stages of infection, by contributing substantially to neutralization and by counteracting ADE. In addition, our results highlight structural differences between strains of Zika and dengue viruses that are used for analyzing infection-enhancement by cross-reactive antibodies. These findings underscore the possible impact of specific antibody patterns on flavivirus disease and vaccination efficacy.

## Author summary

The explosive spread of Zika virus, a flavivirus, to South- and Central America underscores the potential threat of newly emerging arthropod-borne viruses. Zika virus infection can cause congenital birth defects and neurological complications. Many people at risk are flavivirus pre-immune because of prior infections with other flaviviruses (e.g. dengue virus, which co-circulates in Zika outbreak regions) or vaccinations (e.g. against yellow fever or tick-borne encephalitis) and have non-protective cross-reactive antibodies at the time of infection. Since pre-existing immunity can modulate the specificity and functional activity of antibody responses, and cross-reactive antibodies have been implicated in disease enhancement, we compared the specificities of serum samples from flavivirus-naïve and vaccinated individuals after primary Zika virus infections. Prior immunity led to a strong booster of cross-reactive antibodies that did not neutralize Zika virus. Importantly, we could also show that newly formed IgM antibodies contributed significantly to virus neutralization and prevented infection enhancement by other antibodies. Our data thus show how pre-existing cross-reactive immunities can alter the specificities and functional activities of antibody responses in flavivirus infections, which may affect flavivirus-induced disease and the efficacy of vaccinations.

## Introduction

Epidemics of infections with Zika virus, a mosquito-borne flavivirus, first emerged in Pacific islands (Yap 2007, French Polynesia 2013) followed by a big outbreak in South and Central America starting in 2015 [1, 2]. Due to the high number of cases, an association with Guillain-Barré syndrome in adults and severe neurodevelopmental disease in newborns after infection of pregnant women became apparent [3]. Zika virus is a close relative of other important human pathogenic flaviviruses like dengue (DEN) virus, yellow fever (YF) virus, West Nile (WN) virus, Japanese encephalitis virus, tick-borne encephalitis (TBE) virus and Powassan virus. All these viruses are antigenically related and not only induce type-specific and protective antibodies, but also broadly flavivirus cross-reactive, non-protective antibodies [4]. Especially in the case of sequential infections with the four serotypes of dengue viruses, the presence of such cross-reactive antibodies at the time of infection has been implicated to contribute to severe forms of disease [5, 6].

In sequential infections with different flaviviruses, anamnestic responses to shared epitopes can lead to the phenomenon of original antigenic sin, resulting in a strong boost of cross-reactive antibodies at the expense of type-specific antibodies to the newly infecting virus [7, 8]. This situation can occur in regions where two or more flaviviruses co-circulate, such as the four dengue viruses in all tropical and sub-tropical regions around the world or dengue and Zika viruses in South and Central America or Southeast Asia [9, 10], or as a result of immunization with different flavivirus vaccines [11–13]. Pre-existing cross-reactive immunity has thus the potential to affect the antibody response in different combinations of flavivirus infections and vaccinations, which not only includes the modulation of IgG specificities but may also impair the development of newly formed IgM antibodies [14–17]. In our study, we have therefore analyzed the antibody response in groups of individuals with recent Zika virus infections that were either flavivirus-naïve or had previously been vaccinated against TBE and/or YF.

**Fig 1:**
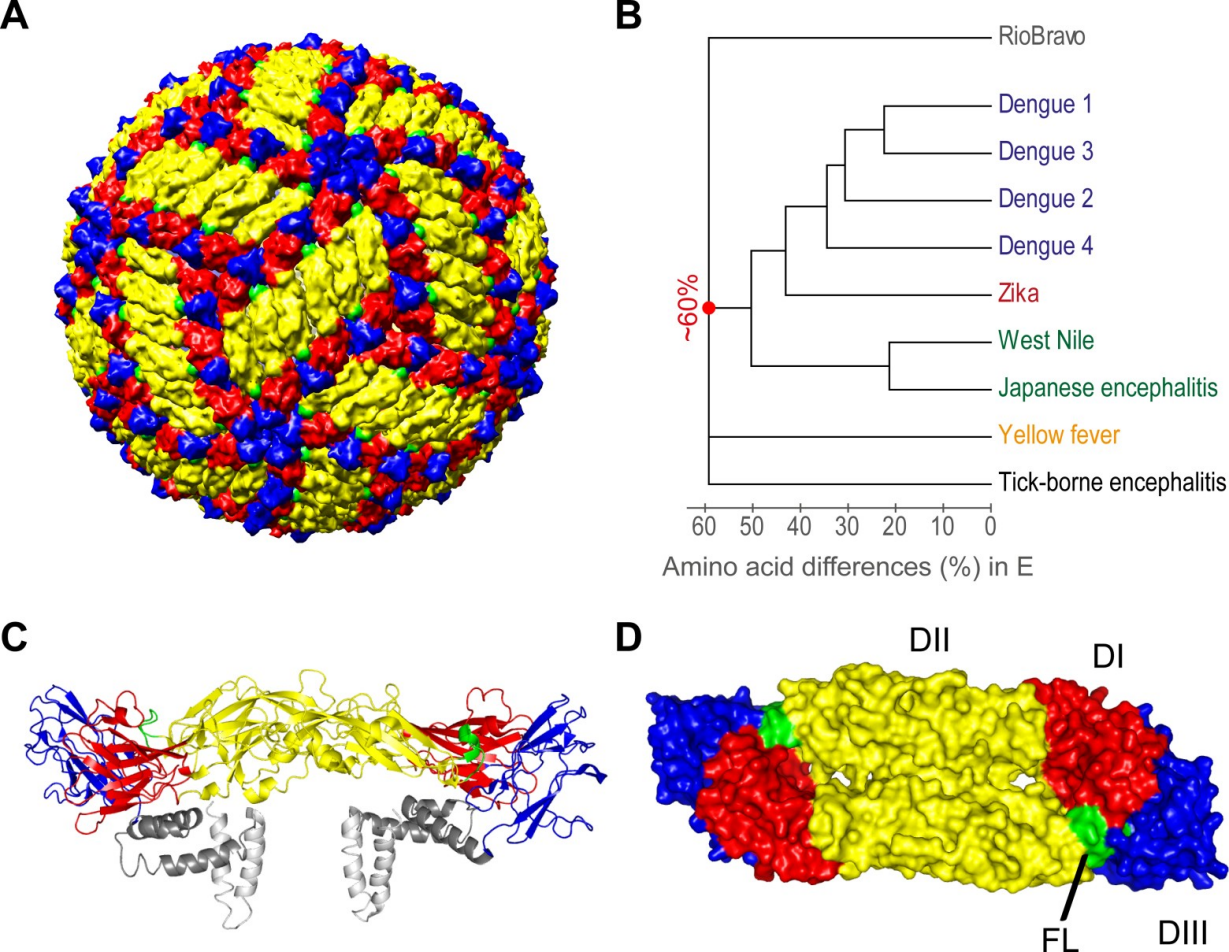
Structure of Zika virus and antigenic relationships of flaviviruses. (A) Zika virus particle (PDB: 6CO8, [22]). (B) Distance relationships between flavivirus E proteins based on amino acid sequence differences. (C) Ribbon diagram of the Zika virus E dimer (side view) and (D) surface representation of the Zika virus E dimer (top view). Color code: domain I (DI), red; domain II (DII), yellow; domain III (DIII), blue; stem and transmembrane domains, gray; fusion loop (FL), green. Figures were prepared with Pymol (Schrödinger LLC, www.pymol.org) and Chimera (http://www.rbvi.ucsf.edu/chimera, [23]). Sequences of E were obtained from the ViPR data bank (https://www.viprbrc.org). GenBank accession numbers are as follows: U27495 for tick-borne encephalitis virus, AY640589 for yellow fever virus, DQ211652for West Nile virus, D90194 for Japanese encephalitis virus, KJ776791 for Zika virus, AF226687 for dengue 1 virus, DQ863638 for dengue 3 virus, M29095 for dengue 2 virus, GQ398256 for dengue 4 virus, and NC_003675 for Rio Bravo virus.

The main target of flavivirus neutralizing and protective antibodies is the envelope protein E [4], which covers the surface of mature virions and forms a herringbone-like icosahedral shell [18] (Fig 1A). Although the amino acid sequences of E diverge up to 60% among distantly related flaviviruses (Fig 1B), the overall structure of this protein is similar in all instances, with an ectodomain that is connected to the transmembrane anchor via a stem region containing two or three helices (Fig 1C). The ectodomain consists of three individual domains (DI, DII, DIII) and forms an antiparallel homodimer (Fig 1D). The fusion loop (FL), located at the tip of DII (Fig 1D), is the most conserved sequence element in E and therefore the major inducer of broadly flavivirus cross-reactive antibodies [19, 20]. In ‘closed-shell’ models of flavivirus particles (Fig 1A), the FL appears to be buried at the dimer interface (Fig 1D). However, flavivirus particles were shown to exhibit dynamic motions (‘virus breathing’) that can lead to the exposure of such cryptic antigenic sites [19, 21].

The immune response to primary flavivirus infections is usually characterized by a rapid rise of specific IgM antibodies, which appear to play an important protective role in the early phase of infection [24, 25]. In sequential flavivirus infections, however, the extent of IgM antibody formation may be reduced and/or delayed [14–17, 26, 27]. Although E-specific IgG antibodies are crucial for maintaining long-lasting flavivirus immunity [21, 28, 29], they can also cause antibody-dependent enhancement (ADE) of infection of Fcγ receptor-positive cells when present at sub-neutralizing concentration [30]. This phenomenon has been demonstrated in vitro as well as in mouse experiments for several pairs of flaviviruses [31, 32]. In human infections, ADE has been linked to a higher frequency of severe forms of dengue in sequential infections with different serotypes [5, 6], even following live vaccination [33, 34].

Since the modulation of antibody responses by pre-existing immunity can have profound biological consequences, we investigated the effect of pre-existing vaccine-induced immunity on IgM and IgG profiles after recent primary Zika virus infection. For this purpose, we analyzed samples from Zika patients, who were either flavivirus naïve or had previously been vaccinated against TBE and/or YF. We demonstrate that virus-specific IgM antibodies contribute substantially to Zika virus neutralization in the early phase of infection and play an important role in the prevention of IgG-mediated enhancement of Zika virus infection of Fcγ receptor-positive cells. Importantly, the formation of IgM antibodies is reduced in individuals with pre-existing cross-reactive flavivirus immunity. Pre-vaccinated individuals displayed a strong anamnestic response of cross-reactive IgG antibodies, which caused ADE of dengue but not of Zika virus infection in vitro. Our findings contribute to the understanding of the complex interplay of antibody populations in transient early stages of flavivirus infections, which can affect the clinical outcome of infections and the efficacy of vaccines.

## Methods

### Ethics statement

Human serum samples from 62 Zika patients were sent to the Center for Virology of the Medical University of Vienna for diagnostic purposes. As negative controls, 32 flavivirus-negative samples from a previous diagnostic study, in which we analyzed TBE antibody responses, were included [35]. The samples were tested anonymously with the approval of the ethics committee of the Medical University of Vienna (EK134/2008). No samples were specifically collected for this study.

### Human serum samples

We analyzed serum samples from 62 patients who were diagnosed with an acute symptomatic Zika virus infection after returning to Europe from Zika-endemic areas. They developed Zika virus-specific IgM and neutralizing antibodies, and 30 patients were Zika PCR positive (23 negative, 9 not tested). Samples from only two of these Zika patients exhibited a low degree of broad flavivirus cross-reactivity of IgM, as revealed by Rio Bravo (RB) and dengue IgM assays. Previous flavivirus immunity (YF and/or TBE vaccinations) was confirmed by neutralization assays (see below); the time points of vaccinations were not known to us. All serum samples were heat-inactivated for 30 min at 56°C prior to serological testing. Pools were prepared from equal aliquots of serum samples.

### Confirmation of immune status by TBE and YF neutralization tests (NTs)

TBE and YF NTs were performed as described previously [26, 36, 37]. Briefly, serial dilutions of serum samples were mixed with TBE virus strain Neudoerfl [38, 39] or YF virus strain 17D [40]. BHK-21 cells (ATCC) were added and incubation was continued for 3–4 days. In the case of YF virus, virus neutralization titers were expressed as the reciprocal of the serum dilution that was required for 100% protection against virus-induced cytopathic effects. Virus neutralization titers ≥20 were considered positive. Since TBE virus does not produce a cytopathic effect, virus replication and its inhibition was determined by measuring the presence of virus in the cellular supernatants using a four-layer ELISA as described previously [41, 42]. The virus neutralization titer was defined as the reciprocal of the serum dilution that gave a 90% reduction in the absorbance readout in the assay compared with the control without antibody. Virus neutralization titers ≥10 were considered positive.

### Recombinant antigens

The flavivirus E proteins used in this study (Table 1) were produced with the Drosophila expression system (Invitrogen) as described previously [40, 43–45]. The expression vector pT389 (kindly provided by Thomas Krey and Felix Rey, Institute Pasteur, France), encoding the E gene lacking the stem-anchor region (synthesized by GeneArt/Thermo Fisher Scientific, Table 1), an enterokinase cleavage site and a double strep-tag (C-terminal), was transfected into Drosophila S2 cells by CaCl_2._ Stably transfected cells were selected with blasticidin (Fisher Bioreagents). After induction of protein expression by CuSO_4_, proteins were affinity purified with Strep-Tactin columns (IBA GmbH, Göttingen, Germany) according to manufacturer’s instructions.

**Table 1:** Recombinant soluble E proteins^a^.

virus	strain	NCBI Nucleotide	NCBI Protein	amino acids of E	reference
Zika virus	H/PF/2013	KJ776791	AHZ13508.1	1–408	[44]
Rio Bravo virus	RiMAR	AF144692.1	AAF37322.1	1–393	[45]
TBE virus	Neudörfl	U27495	AAA86870.1	1–400	[40, 43]
Dengue virus 2	16681	NC_001474.2	NP_056776.2	1–399	this study
WN virus	NY99	AF196835	AAF20092.2	1–400	[43]

^a^ All recombinant E proteins lack the stem-anchor region and contain a C-terminal strep-tag

Purified proteins were quantified with the Pierce BCA Protein Assay Kit (BCA, bicinchoninic acid), (Thermo Fisher Scientific) according to manufacturer’s protocol. Purity was verified with 15% SDS-PAGE according to Laemmli [46] and/or Agilent 2100 Bioanalyzer electrophoresis (Agilent Technologies Inc.).

### Determination of the oligomeric state of the recombinant Zika E protein

Zika and WN virus E proteins were analyzed by rate zonal ultracentrifugation in sucrose gradients as described previously [43, 47]. The monomer control for the Zika virus E protein was prepared by incubation with 1% SDS for 30 min at 65°C. 3–5 μg E protein were applied to the gradients and centrifuged for 20 h at 38,000 rpm at 15°C in a Beckman SW40 rotor. The gradients were fractionated and the amount of antigen in each fraction was quantified by ELISA using Strep-Tactin-coated plates for capturing the strep-tagged antigen and the broadly cross-reactive FL-specific monoclonal antibody (mab) A1 [48] together with rabbit anti-mouse IgG (H+L)-horse radish peroxidase (HRP) (Nordic Biosite). E protein was quantified using the corresponding purified protein as a standard.

Size-exclusion chromatography of the Zika and WN virus E proteins was carried out with a Superdex 200 Increase 3.2/300 column using the ÄKTA FPLC system (GE Healthcare) according to the manufacturer’s instructions in TAN buffer pH 8.0.

### Flavivirus IgG ELISA

25 ng/well strep-tagged E protein were added to Strep-Tactin-coated microplates (IBA GmbH, Göttingen, Germany) and incubated for 1.5 h at 37°C in phosphate-buffered saline (PBS) pH 7.4, 2% sheep serum, 2% Tween 20. After blocking with 1% bovine serum albumin (BSA) in PBS pH 7.4, serial dilutions of samples were added and incubated for 1 h at 37°C. Serial dilutions of bound human antibodies (mabs, polyclonal serum samples) were detected with goat anti-human IgG labeled with HRP (Thermo Fisher Scientific). The starting dilution for ELISAs with single serum samples was 1:100 and that with pools 1:300. Absorbance was measured at 450 nm. Titers were determined by curve fitting with a four-parameter logistic regression with GraphPad Prism 8 (GraphPad Software Inc.). A positive control serum was included on each ELISA plate. This control serum was obtained from a Zika patient with YF and TBE pre-immunity and yielded similar titration curves with Zika, RB, TBE and dengue serotype 2 virus E proteins (S1 Fig).

Validation of the ELISAs was performed as follows: Proper display of the antigens in this ELISA format was verified by the use of a number of E-specific monoclonal antibodies (mabs). These included FL-specific antibodies (A1, 4G2 and B12 –[44, 48–50], giving a positive reaction in all assays), E dimer-dependent (EDE)-antibodies (C8 and C10 –[44, 50], giving a positive reaction in Zika and dengue ELISAs), the dengue-specific mab 4E11 [51], and TBE-specific mabs A3, A4, A5, B2, IC3 [52] (giving a positive reaction in the TBE ELISA only). For cut-off determination of the Zika and RB assays, we used 32 diagnostic serum samples that had proven to be TBE-negative in a previous study [35]. All of these controls were re-evaluated and confirmed to be negative in IgG ELISAs with E proteins from Zika, Rio Bravo, TBE and dengue serotype 2 viruses (S1 Fig). The cut-off for titer determinations was set as the mean absorbance value from these negative controls at a 1:100 dilution plus 3 standard deviations.

### Zika IgG avidity ELISA

Relative avidities of polyclonal serum samples were analyzed with Zika virus E protein in the flavivirus IgG ELISA described above including a short exposure to urea as performed in standard virus diagnostic assays [53]. Strep-Tactin XT plates were used for capturing the antigen as recommended by the manufacturer (IBA GmbH, Göttingen, Germany) to avoid elution of the strep-tagged E protein from Strep-Tactin-coated plates by urea. After incubation of serum samples with the antigen, the plates were incubated for 5 minutes at room temperature (RT) with and without 6M urea (Biorad, reagent for protein electrophoresis) in PBS pH 7.4 [54, 55]. Urea was added to the buffer immediately before use and did not change its pH. After the urea incubation step, the plates were washed twice with PBS pH 7.4 before the assay was completed and evaluated for titer determination as described for the flavivirus IgG ELISA. Relative avidities were calculated according to the following formula: Avidity (%) = (titer plus urea/ titer without urea) x 100. The performance of the assay was controlled using serum samples obtained early and late after Zika virus infection (S2 Fig).

### Zika and RB IgM ELISA

MaxiSorp microtiter plates (Nunc) were coated with 28 ng/well polyclonal rabbit anti-human IgM (Dako) in carbonate buffer pH 9.6 [35]. Diluted human serum samples were added for 45 min at 37°C. Bound IgM antibodies were then detected with the strep-tagged E proteins and Strep-Tactin-labeled HRP (IBA GmbH, Göttingen, Germany). For the quantitative Zika IgM ELISA, a Zika virus post-infection serum sample (TBE-pre-vaccinated patient) was included as a standard, set at 1,000 IgM arbitrary units (S3 Fig). Quantification of unknown samples was performed with dilutions within the linear range of the standard serum. The cut-off (75 IgM units) was determined with 32 flavivirus negative samples (S3 Fig). In the case of the RB IgM ELISA, the cut-off was calculated as the mean absorbance value of the negative samples plus 3 standard deviations.

### Zika, TBE and dengue neutralization tests (NTs)

NTs were performed with Zika virus strain H/PF/2013 (European virus archive), dengue virus serotype 1 strain Hawaii (kindly provided by Herbert Schmitz, Bernhard-Nocht-Institute), dengue virus serotype 2 strain NGC (European Virus Archive) and TBE virus strain Neudoerfl in black 96-well microtiter plates (Greiner Bio-One). The assays were carried out in principle as described previously for YF virus [40], but fluorescence-labeled instead of alkaline phosphatase-labeled secondary antibodies were used for detection. In each assay, a positive and negative polyclonal serum were included as controls. Briefly, serially diluted serum samples were incubated with a pre-determined dilution of virus for 1 h at 37°C. Then BHK-21 (TBE virus) or Vero cells (ECACC) (Zika virus, dengue virus) were added and incubated for 3 (Zika virus, TBE virus) or 4 (dengue virus) days. After removal of cell culture supernatants, the cells were fixed with 4% paraformaldehyde for 20 min at RT, and incubated with a Tris-buffer (50 mM Tris, 150 mM NaCl, pH 7.6) containing 3% nonfat dry milk, 0.5% Triton X-100, and 0.05% Tween 20 for 30 min at 37°C. Mouse monoclonal antibodies recognizing viral structural proteins (Table 2) were then added and the cells were incubated for 1.5 h at 37°C. Bound antibodies were detected with a rabbit-anti-mouse-Alexa Fluor 488-labeled secondary antibody (Invitrogen). Fluorescence was measured with Synergy HTX (excitation: 485/20 nm, emission: 528/20 nm, Biotek). Titers were determined after curve fitting with a four-parameter logistic regression using 50% of the fluorescence in the absence of antibody as a cut-off (NT_50_).

**Table 2:** Monoclonal antibodies used as detectors in NTs.

virus	mab	reactivity	reference
dengue virus	2H2 (ATCC HB114)	dengue prM	[56, 57]
TBE virus	IC3	TBE E DI	[58]
Zika virus	A1	flavivirus FL-specific	[48]

The assays were validated with neutralizing mabs specific for the different viruses, as follows: Zika and dengue viruses were neutralized by the EDE-specific mabs C8 and C10, corresponding to their activities reported in the literature [44, 50]. Dengue viruses were also tested with the dengue-specific mab 4E11 [51]. TBE virus neutralization was controlled with the type-specific mab A3 reacting with E domain II [52].

### Antibody-dependent enhancement (ADE) assay

Serial dilutions of heat-inactivated serum samples were pre-incubated with either Zika virus or dengue virus serotype 2 (5,000–10,000 viral RNA copy numbers) for 1 h at 37°C. Fcγ receptor-bearing K562 cells (ATCC) were added and incubated for 3 (Zika virus) or 4 (dengue virus) days. Then the cell culture supernatant was harvested and used for virus detection by qPCR. Viral RNA was isolated with the RNeasy Mini Kit (Qiagen) and cDNA was generated with the iScript cDNA Synthesis Kit (BioRad), according to manufacturers’ instructions. cDNA was mixed with TaqMan Universal PCR Master Mix (Applied Biosystems/Thermo Fisher Scientific), 25 pmol of primers and 10 pmol of probe (Eurofin Genomics). Primers and probes used in this study targeted the NS5 gene: Zika virus: forward primer: ACAAGGGGAATTTGGAAAGGC; reverse primer: GAATCCAAGGGCTTCGAACTC; probe: FAM-AGCCGCGCCATCTGGTATATGTGG-TAMRA. Dengue virus 2: forward primer: CAGATGGAGGGAGAAGGAGTC; reverse primer: CGCCCTACTCTTGCTAACCA; probe: FAM-ACAGTCACAGAAGAAATCGCCGTGCA-TAMRA. The samples were amplified under the conditions described in [59]: 3 min at 50°C followed by 10 min at 95°C, and 45 cycles of 95°C (15 s), 55°C (30 s), and 72°C (30 s) using Quantstudio 3 (Thermo Fisher Scientific). A plasmid encoding part of the NS5 gene of Zika virus or dengue virus 2 (synthesized by GeneArt/Thermo Fisher Scientific) was included to generate a standard curve with 10-fold dilutions for quantification, as described previously [59, 60]. The first point of the linear standard curve was 10^8^ RNA copies/reaction, the last point 100 RNA copies/reaction (limit of detection, because this amount resulted consistently in detectable PCR amplification above background levels). Fold enhancement of infection was calculated as the ratio between viral RNA copies in the presence of a positive serum and viral RNA copies in the presence of a flavivirus-negative control serum.

ADE was validated with mab 4G2, which caused slight ADE of dengue virus infection as described in the literature [61], and we observed no enhancement of Zika virus under these conditions. As a negative control for both viruses we used mab E24, specific for the WN virus E protein [62].

### Depletion of antibodies with Zika or RB virus E protein

For the depletion of Zika virus-specific or flavivirus broadly cross-reactive antibodies, 200 μg Zika or RB virus E protein were bound to Strep-Tactin XT spin columns (IBA GmbH, Göttingen, Germany), equilibrated with binding buffer (50mM NaH_2_PO_4_, 300 mM NaCl, 0.05% Tween 20, pH 8.0). Columns were spun down for 30 s at 700 g and the flow-through was reloaded six times. After a wash step with binding buffer, the column was equilibrated with depletion buffer (PBS pH 7.4, 0.1% BSA). Pre-diluted serum samples (1:10–1:15) were loaded onto the columns and incubated for 10 min at RT. To deplete completely Zika or RB virus-reactive antibodies a minimum of eight rounds was necessary. Depletion was verified by IgG ELISA with the respective E protein as antigen as described above. Mock depletion was performed with Strep-Tactin XT spin columns in the absence of E protein.

### IgG depletion

IgG depletions were performed with Ab SpinTraps (GE Healthcare) containing Protein G Sepharose following the manufacturer’s instructions. For mock depletions, empty spin columns (Thermo Fisher Scientific Pierce) were loaded with Sepharose 4B beads (Sigma). Columns were equilibrated with binding buffer (20 mM Na_2_HPO_4_, pH 7.0) and centrifuged for 30 s at 100 g and 30 s at 300 g. 100 μl of 1:2 pre-diluted serum samples were loaded onto the columns and incubated for 75 min at RT under gentle shaking. The columns were then centrifuged for 30 s at 100 g. The IgG depleted eluate was collected and the column was washed twice with 75 μl binding buffer. The eluates were pooled resulting in an IgG-depleted serum with a final dilution of 1:10. Depletion was verified by the flavivirus IgG ELISA (described above), using the Zika virus E protein, and the presence of IgM in the final eluate was confirmed by Zika IgM ELISA (described above).

### IgM depletion

1 ml anti-IgM agarose beads (Sigma) were mixed with 2 ml binding buffer (PBS, 1% BSA, pH 7.4) and were centrifuged for 5 min at 4,000 g. The supernatant was removed and the beads were washed with 2 ml binding buffer. Mock depletions were performed with Sepharose 4B beads (Sigma). Serum was diluted 1:2 in binding buffer and added to the beads. The mixture was incubated for 90 min at RT under gentle shaking. After centrifugation for 5 min at 4,000 g, the supernatant was subjected to control ELISAs. Depletion was verified by Zika IgM ELISA (described above), and the presence of IgG was confirmed by the flavivirus IgG ELISA (described above), using the Zika virus E protein.

### Statistics

Antibody titers that were less than the lower detection limit of the assay were assigned values equal to 50% of this limit for data analysis. IgG ELISA titers and NT_50_ titers <100 were therefore considered 50.

Titers were log-transformed for statistical analysis. Antibody titers and units of pools were analyzed by one-way ANOVA with Dunnett’s post-hoc test (GraphPad Prism 8). All other statistics were performed with SPSS 25.0.

Statistical comparisons of pool and single serum data of the analyses were performed by applying a general linear model (GLM) with the log-link function (S1 Table).

Comparisons of the four pools (naïve, YF, TBE, and YF+TBE) were done by application of the GLM with data from naïve pools as reference. Maximum likelihood estimates of parameters reflecting the difference to naïve pools were tested for significance by Wald’s tests. Deviations of residuals from normality were assessed by Shapiro-Wilk’s tests.

Comparisons of mock versus real depletion within pools (naïve, YF, TBE, and YF+TBE) were performed by applying a two-factor Analysis of Variance (ANOVA) and linear contrasts.

Relative IgG avidity of pre-immune sera against naïve ones were tested by comparing the reduction of log titers using the sera untreated with urea as offset applying a GLM.

Fold enhancement of Zika and dengue virus infectivity between depleted and mock-depleted sera was compared by computing the area under the log-dilution-log-fold-enhancement curves using GLM.

Fold enhancement of Zika virus between depleted and mock-depleted sera (using a single serum dilution) was compared by applying a GLM with linear contrasts and a Bonferroni-Holm adjustment.

Trends of antibody responses (individual serum samples) were tested by fitting a polynomial function to the data and comparing the slopes of the linear component as well as the maximum of the response reached during the observation period using GLM.

For all analyses, a p-value below 0.05 was considered significant.

## Results

### Study population

To assess the effect of pre-existing flavivirus immunity on the patterns of antibody responses in primary Zika virus infections, we analyzed serum samples from laboratory-confirmed Zika patients (travelers returning from Zika-endemic regions) with and without prior TBE and/or YF vaccination (Table 3). Since these samples were not derived from a prospective study but were randomly received for diagnostic purposes from travelers returning with Zika virus infections, the groups were relatively heterogeneous with respect to age and comprised mostly adults. Flavivirus pre-immunity due to vaccination was confirmed by TBE and YF neutralization assays as described in Methods. The time points of vaccinations, however, have not been documented for the diagnostic samples used.

**Table 3:** Zika patients.

flavivirus pre-immunity	patients n	median age in years (range)	sex f/m	samples n^a^
naïve	16	38 (15–54)	9/7	24
pre-immune	46	37 (18–71)	22/24	81

^a^ For some patients multiple samples were available.

Our analyses included the quantification of Zika virus-specific and flavivirus cross-reactive IgM and IgG antibodies in ELISA and NT, as well as the determination of in vitro ADE of Zika and dengue virus infections. Because these experiments required relatively large volumes for the depletion of antibody subsets (IgM, IgG, cross-reactive antibodies), these analyses were first carried out with pools of equal aliquots of serum samples (one sample per patient) for which at least 500 μl were available. The number of patient samples included in these pools from the different groups are shown in Table 4, with the YF pool (n = 4) being disproportionally smaller than the other three groups (n = 9 or 10) (Table 4). For this reason, we used 300 μl aliquots for the YF pool, and 200 μl aliquots for the three other pools.

**Table 4:** Serum samples from flavivirus naïve and pre-immune Zika patients used for pools.

flavivirus pre-immunity		patients n^a^	median age in years (range)	sex f/m	days after onset median (range)
	vaccination				
naïve	none	10	33 (24–54)	8/2	33 (21–60)
pre-immune	TBE	10	36 (18–39)	5/5	36 (22–71)
	YF	4	61 (51–71)	1/3	34 (30–49)
	TBE and YF	9	41 (21–68)	5/4	40 (25–65)

^**a**^ One sample per patient was included in the pools.

### Characterization of the Zika virus E dimer

For the analysis of Zika virus-specific antibodies, we used a recombinant Zika virus E protein with a C-terminal strep-tag and lacking the stem-anchor region (see also Fig 1C and 1D). This protein crystallized as a dimer in the absence and presence of mabs recognizing an epitope that is dependent on the dimeric structure of E (E-dimer dependent epitope, EDE) [44]. In addition, the Zika virus E protein was shown to have high affinity for an EDE-specific mab by Biolayer Interferometry [44].

The stable dimeric structure of the Zika virus E protein in solution was verified by sedimentation analyses and size exclusion chromatography in comparison to a recombinant WN virus E protein (S4 Fig), which was shown to be a monomer, both in solution as well as in crystals [54, 63, 64].

In addition, we analyzed whether our Zika E dimer is recognized by the human EDE-specific mabs C8 and C10 [44] in the capture ELISA format used for all IgG determinations in this study. The protein was bound via its strep-tag to Strep-Tactin-coated plates (Methods) and exhibited excellent reactivity with mabs C8 and C10 (S4 Fig), demonstrating that dimer-specific epitopes are presented under the assay conditions used. To further analyze the functional activity of polyclonal antibodies detected with the Zika virus E dimer, we depleted three of the four pools listed in Table 4 as well as four serum samples from individual Zika patients (1 serum from each pool) with this protein. As shown in S5 Fig, approximately 90% or more of NT activity was removed, indicating that the dimeric Zika antigen allows the measurement of most of the functionally important antibodies.

### Antibody determinations of serum pools and individual sera

**Fig 2:**
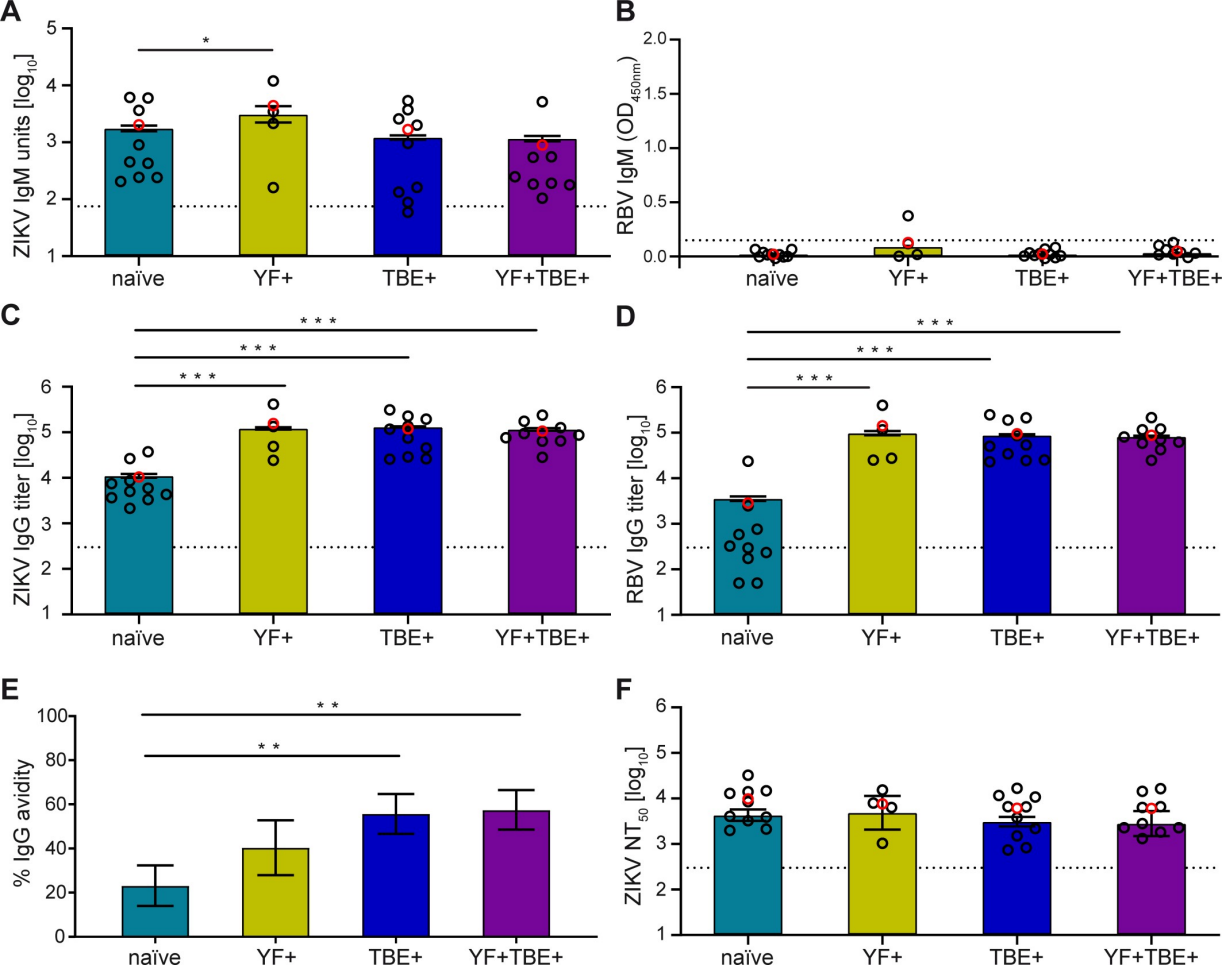
Zika virus-specific and broadly cross-reactive antibody titers of four serum pools (colored columns) and the individual serum samples of these pools (black circles). The mean values of individual sera are shown as red open circles. (A) Zika virus IgM ELISA units, (B) Rio Bravo virus IgM ELISA absorbance values (cross-reactive antibodies), (C) Zika virus IgG ELISA titers, (D) Rio Bravo virus IgG ELISA titers (cross-reactive antibodies), (E) relative Zika virus IgG avidities (%), (F) Zika virus NT_50_ titers. The cut-off is indicated by a dotted line. Mean values were calculated from at least three independent experiments except panel B (two independent experiments). Error bars represent the standard error of the mean (SEM; A, C-F) or the range (B). The statistics shown in the figure refer to the pool data. Statistical comparisons of pool data and mean values of individual sera are given in S1 Table. (A, C, D, F): One-way ANOVA followed by Dunnett´s test, (E): General linear model and Wald’s test. Significant differences between the naïve pool and the pre-immune pools are indicated; *, p < 0.05; **, p < 0.01; ***, p < 0.001. ZIKV, Zika virus; RBV, Rio Bravo virus; naïve, flavivirus naïve serum pool; YF+, yellow fever pre-vaccinated serum pool; TBE+, tick-borne encephalitis pre-vaccinated serum pool; YF+TBE+: yellow fever and tick-borne encephalitis pre-vaccinated serum pool.

The four serum pools and individual sera (see Table 4) were tested in Zika and Rio Bravo (RB) IgM as well as IgG ELISA, and Zika NT. In all cases, the mean values of the pools were similar to those obtained with individual sera that were included in the pool (Fig 2) and comparative statistical evaluation is presented in S1 Table. The comparison revealed a significant difference between pool and individual serum data in only a single case (the naïve pool in RB IgG ELISA), due to an upward distortion caused by one of the individual samples. Without this distortion, the difference between the naïve and pre-immune pools would be even more pronounced. Zika IgG avidity could only be performed with the pools because of volume limitations of individual sera. All four groups produced high amounts of Zika virus-specific IgM antibodies, which were slightly higher in the YF-pre-vaccinated group (Fig 2A). None of the pools contained detectable amounts of broadly flavivirus cross-reactive IgM antibodies as shown in an ELISA with the E protein of the distantly related RB virus (Fig 2B), which shares only approximately 40% of its amino acids with human pathogenic flaviviruses (Fig 1B). In contrast to the IgM results, the Zika virus-specific IgG response was significantly higher in all pre-vaccinated groups than in the naïve group (Fig 2C). This difference was even more pronounced in the RB ELISA (Fig 2D), indicating a larger proportion of broadly flavivirus cross-reactive antibodies in the pre-immune groups. As also suggested by the higher relative avidities of these groups in Zika IgG ELISA (Fig 2E), this pattern is consistent with an anamnestic booster response of IgG in the pre-vaccinated individuals. In the Zika neutralization test (NT), however, no differences were found among the four groups (Fig 2F), showing that the broadly cross-reactive antibodies did not contribute significantly to the neutralization of Zika virus.

### Role of IgM in Zika virus NT and ADE

**Fig 3:**
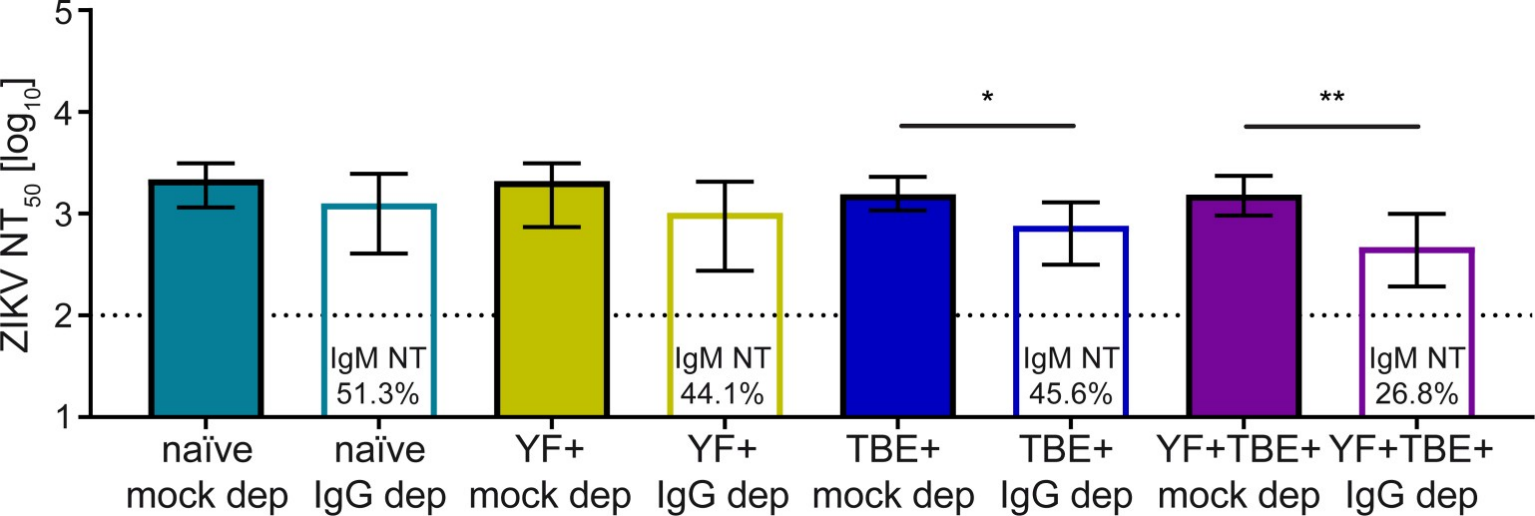
Zika virus neutralization NT_50_ titers by IgM after IgG depletion from serum pools. Colored columns: mock depleted pools containing IgM and IgG; empty columns: IgM only after IgG-depletion. NT titers are shown as mean values +/- SEM from three independent experiments. Numbers in the empty columns represent the means of percent IgM-mediated neutralizing activity (IgM NT) derived from three independent experiments. The dotted line indicates the cut-off of the assay. Asterisks indicate significant differences between the NT_50_ titers of mock and IgG-depleted pools (2-way ANOVA with linear contrasts); *, p < 0.05; **, p < 0.01. ZIKV, Zika virus; naïve, flavivirus naïve serum pool; YF+, yellow fever pre-vaccinated serum pool; TBE+, tick-borne encephalitis pre-vaccinated serum pool; YF+TBE+: yellow fever and tick-borne encephalitis pre-vaccinated serum pool; mock dep, mock-depleted serum pool; IgG dep, IgG-depleted serum pool.

To obtain information about the contribution of IgM antibodies to virus neutralization, we depleted IgG antibodies from the four serum pools using protein G columns (Methods). Control experiments demonstrated that IgG depletion was >99.5% complete (S6A Fig) and no substantial loss of IgM antibodies occurred during the procedure (S6B Fig). As shown in Fig 3, the contribution of IgM to virus neutralization was high in the naïve and single-vaccinated groups, accounting for approximately half of the total neutralizing activity with no significant difference between these groups (p = 0.759 and 0.616). In contrast, in the TBE and YF double-vaccinated group, this contribution was significantly lower than in the naïve group (26.8% vs. 51.3%; p = 0.022). The significance of these differences was calculated by applying a GLM and Wald’s test as described in Methods.

**Fig 4:**
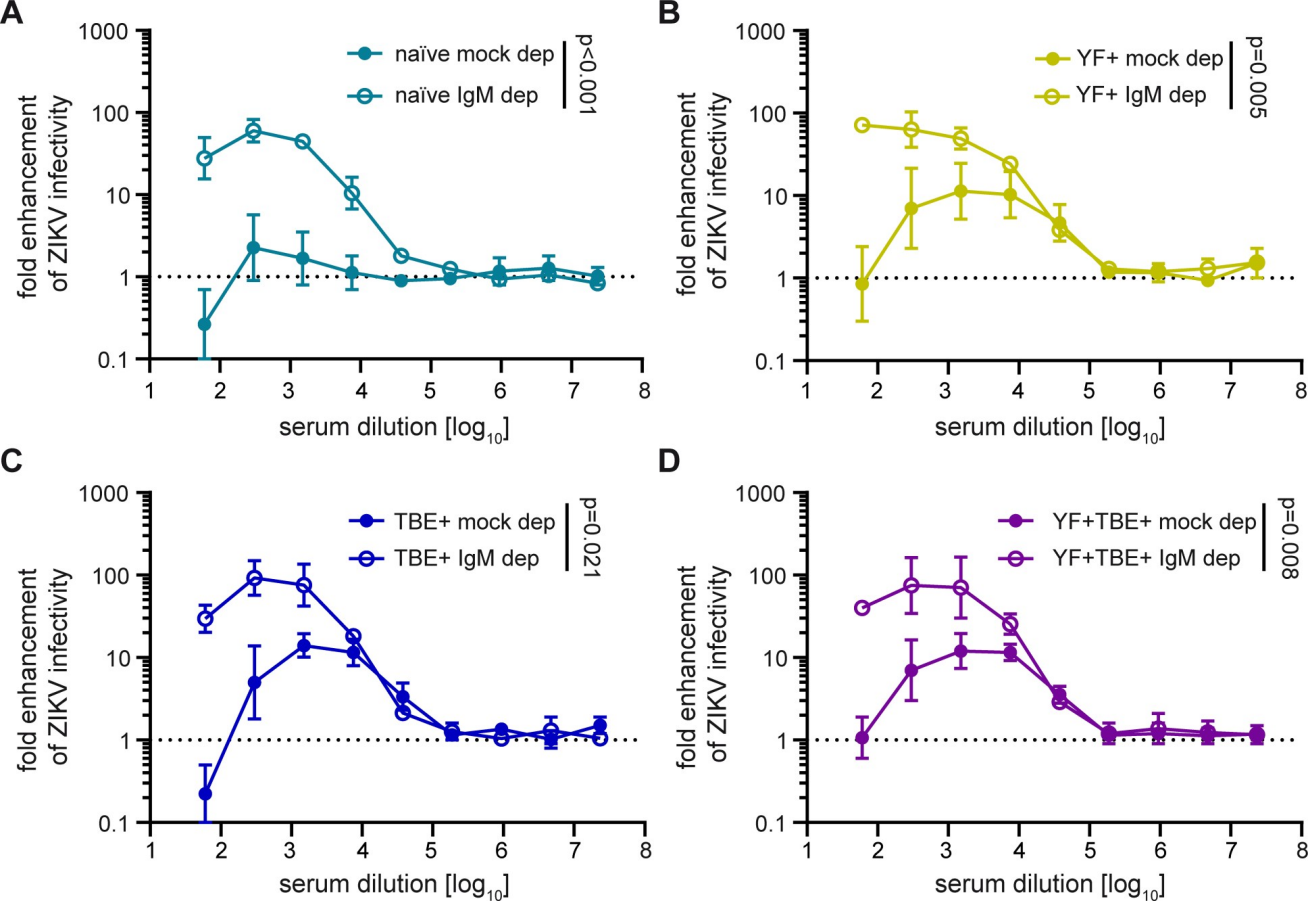
Effect of IgM depletion from serum pools on ADE of Zika virus infection. Serum pools were depleted with IgM agarose beads as described in Methods. Fcγ receptor-positive K562 cells were infected with Zika virus in the presence of serially diluted (A) flavivirus-naïve pool, (B) YF pre-vaccinated pool, (C) TBE pre-vaccinated pool, and (D) YF and TBE pre-vaccinated pool. Colored symbols: mock depletion, empty symbols: IgM depletion. Data are shown as mean values +/- range from two independent experiments. Dotted line indicates no enhancement of infection. Comparison of fold-enhancement of Zika virus infection in the presence of depleted and mock-depleted pools was performed by applying a GLM as described in Methods. p-values are indicated in the graphs. ZIKV, Zika virus; naïve, flavivirus naïve serum pool; YF+, yellow fever pre-vaccinated serum pool; TBE+, tick-borne encephalitis pre-vaccinated serum pool; YF+TBE+: yellow fever and tick-borne encephalitis pre-vaccinated serum pool; mock dep, mock-depleted serum pool; IgM dep, IgM-depleted serum pool.

Since increased infection of Fcγ receptor-positive cells is the result of an interplay between neutralizing and enhancing effects of antibody populations present in a given serum sample [19], we also analyzed to which extent the presence of neutralizing IgM antibodies could affect IgG-mediated Zika virus ADE of infection. For this purpose, we determined the ADE profiles of the four serum pools before and after IgM depletion (Fig 4A–4D), which led to a slight loss of IgG antibodies (S7 Fig). In all cases, removal of IgM antibodies resulted in an increase of ADE, which was statistically significant (Fig 4) despite the relatively strong variation observed for the mock-depleted controls. Using the area under the curve, this difference was highest in the naïve group (1,790-fold), and ranged from 78- to 131-fold in the pre-immune groups, indicating that the presence of neutralizing IgM antibodies had a strong suppressive effect on IgG-mediated ADE in all instances.

**Fig 5:**
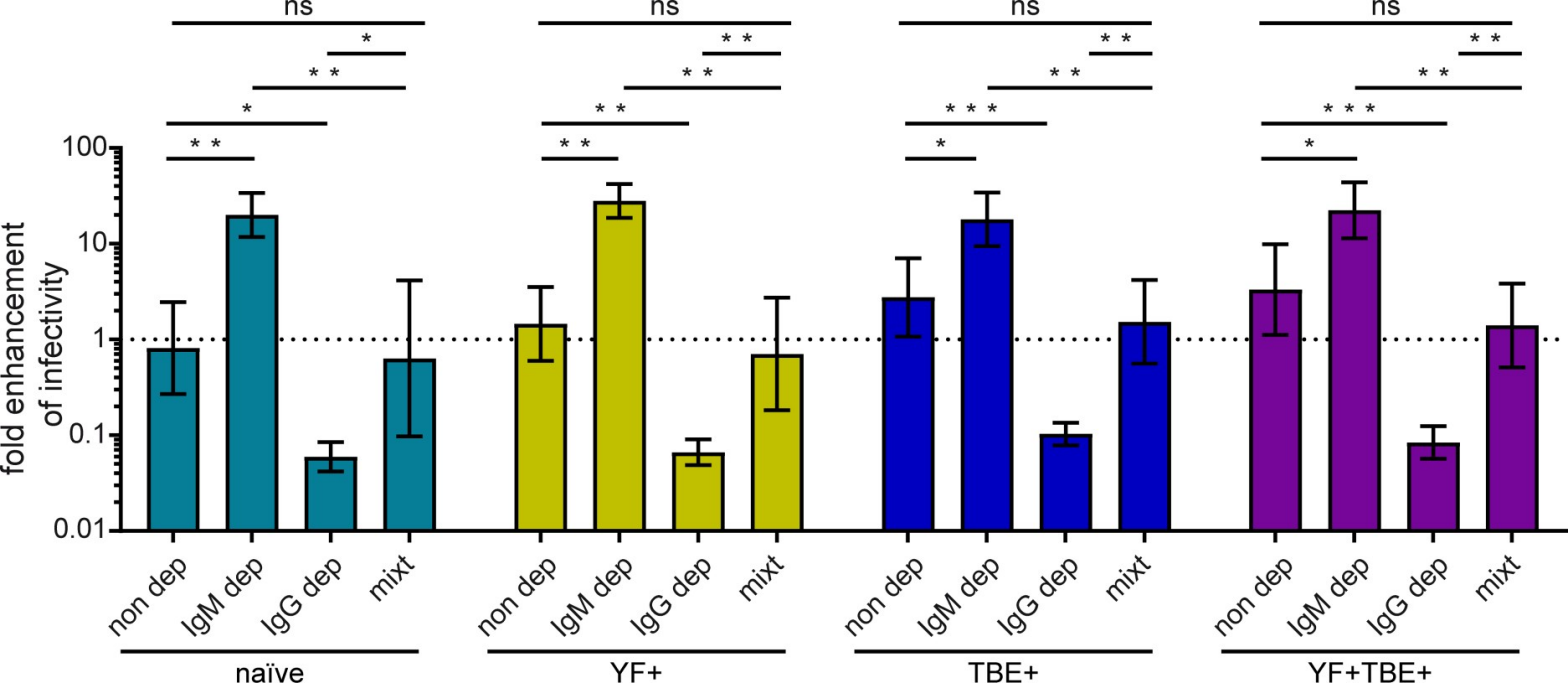
Effect of IgM and IgG depletion on ADE of Zika virus infection. The four serum pools were depleted from IgM or IgG as described in Methods. Fcγ receptor-positive K562 cells were infected with Zika virus in the presence of a 1:1000 dilution of each pool, either non-depleted (non dep), IgM depleted (IgM dep), IgG depleted (IgG dep) or a reconstituted sample, i.e. a mixture of the IgM and IgG-depleted pools (mixt). Data are shown as mean values +/- SEM from three independent experiments. Dotted line indicates no enhancement of infection. Statistical comparisons (with the original and reconstituted samples) were performed by applying a GLM with linear contrasts and a Bonferroni-Holm adjustment and are indicated at the top of the figure. ns, not significant; *, p < 0.05; **, p < 0.01; ***, p < 0.001. Naïve, flavivirus naïve serum pool; YF+, yellow fever pre-vaccinated serum pool; TBE+, tick-borne encephalitis pre-vaccinated serum pool; YF+TBE+: yellow fever and tick-borne encephalitis pre-vaccinated serum pool.

To confirm these results, we carried out additional ADE experiments with single dilutions of the following preparations of the four pools: i. unmodified, ii. IgG depleted, iii. IgM depleted, iv. mixture of equal aliquots of IgG- and IgM-depleted pools (Fig 5). The results show clearly that IgG are responsible for ADE, that IgM have neutralizing activity under the assay conditions, and that the addition of IgM to the IgM-depleted pools counteracted IgG-mediated ADE. Taken together, our data point to the ability of IgM in reducing possible infection-enhancing effects of IgG antibodies early after infection.

### Role of broadly flavivirus cross-reactive antibodies in Zika virus NT and ADE

**Fig 6:**
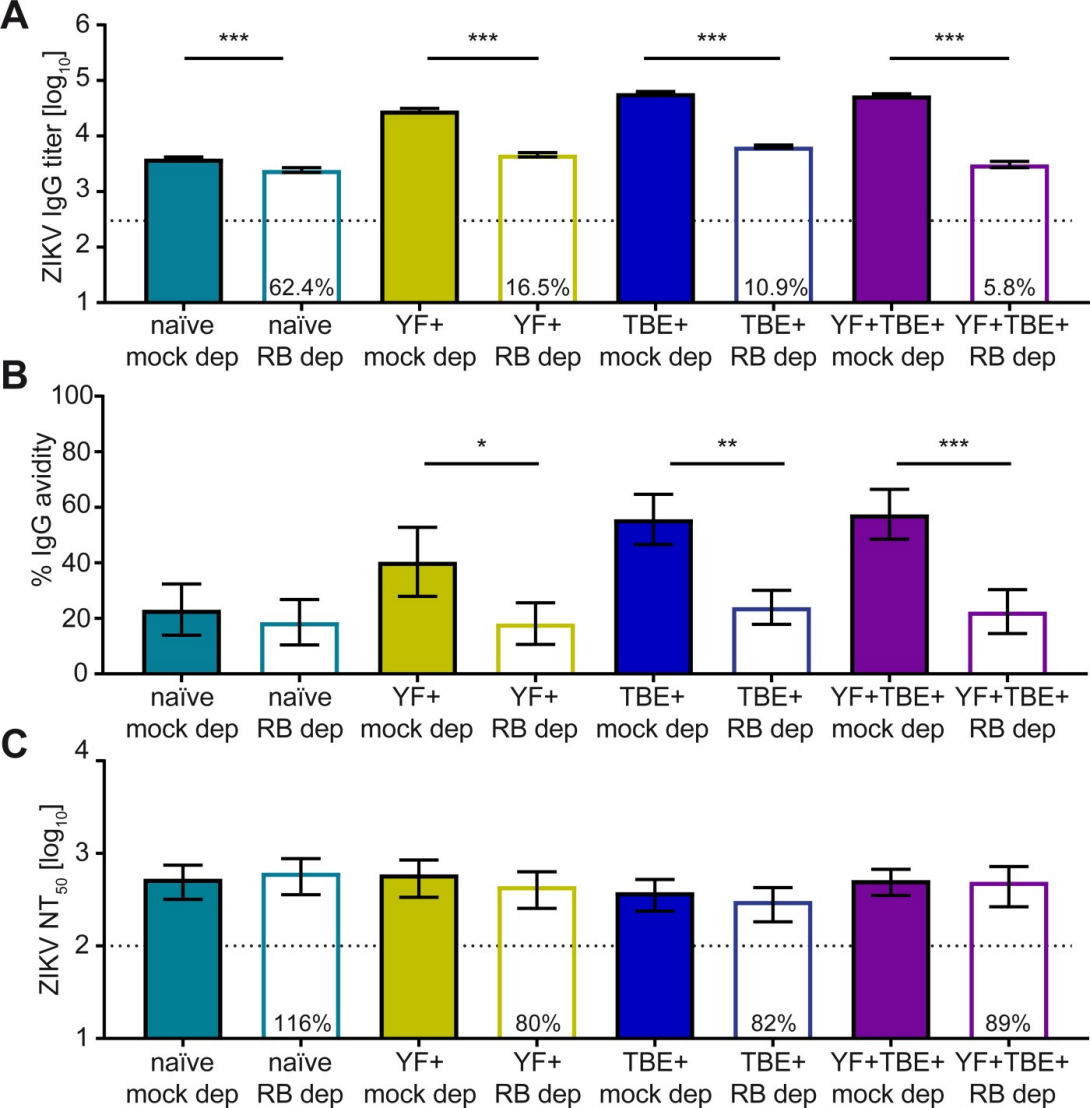
Effect of depletion of cross-reactive antibodies from serum pools with Rio Bravo virus E on Zika virus antibody titers. (A) Zika virus IgG ELISA titers, (B) relative avidity of Zika virus-specific IgG, and (C) Zika virus NT_50_ titers. Colored columns: mock depletion, empty columns: RB depletion. Percentages in the empty columns of (A) and (C) indicate residual ELISA reactivity and neutralizing activity after depletion, respectively. Data are shown as mean values +/- SEM from three independent experiments. The dotted line indicates the cut-off of the assay. Significant differences between mock depletion and RB depletion are indicated by asterisks (2-way ANOVA with linear contrasts); *, p < 0.05; **, p < 0.01; ***, p < 0.001. ZIKV, Zika virus; mock dep, IgM and mock-depleted serum pool; RB dep, IgM and Rio Bravo virus E-depleted serum pool.

**Fig 7:**
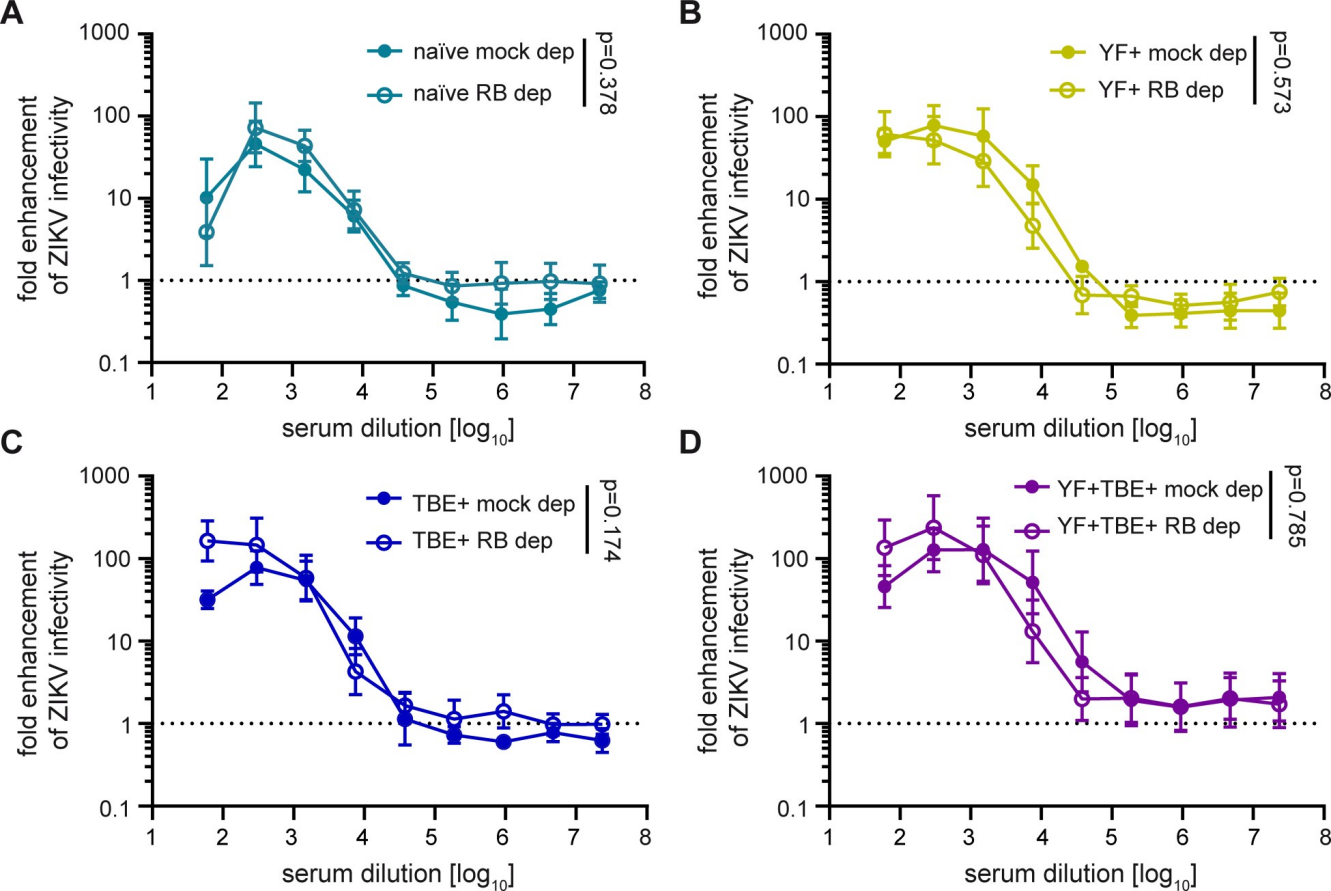
Effect of depletion of cross-reactive antibodies from serum pools with Rio Bravo virus E on ADE of Zika virus infection. Fcγ receptor-positive K562 cells were infected with Zika virus in the presence of serially diluted (A) flavivirus-naïve pool, (B) YF pre-vaccinated pool, (C) TBE pre-vaccinated pool, and (D) YF and TBE pre-vaccinated pool. IgG-containing serum pools were further depleted with RB virus E protein as described in Methods. Colored symbols: mock depletion, empty symbols: RB depletion. Data are shown as mean values +/- SEM from three independent experiments. Dotted line indicates no enhancement of infection. Comparison of fold-enhancement of Zika virus infection in the presence of depleted and mock-depleted pools was performed by applying a general linear model as described in Methods. p-values are indicated in the graphs. ZIKV, Zika virus; mock dep, IgM and mock-depleted serum pool; RB dep, IgM and Rio Bravo virus E-depleted serum pool.

Since the pre-immune groups had significantly higher titers of broadly flavivirus cross-reactive antibodies (Fig 2D), we quantified their contribution to Zika virus neutralization and enhancement by depletion of these antibodies using the RB E protein. As shown by RB ELISA, cross-reactive antibodies were completely removed by this procedure (S8 Fig). Analysis of the depleted samples in Zika IgG ELISA (Fig 6A) revealed that more than 60% of IgG reactivity were Zika virus-specific in the naïve group, contrasting to only 5.8 to 16.5% in the pre-immune groups, in which most of the total virus-specific IgG was broadly cross-reactive (94.2% to 83.5%). Avidity measurements before and after depleting cross-reactive antibodies with the RB E protein (Fig 6B) revealed a significant reduction in all pre-immune groups but not in the naïve group. The data show that the newly synthesized Zika virus-specific antibodies (i.e. those not removed by RB depletion) had a low relative avidity in all groups (empty columns in Fig 6B) with no significant differences, as determined by a GLM and Wald’s test as described in Methods (p = 0.556, 0.756, and 0.998). Depletion of cross-reactive antibodies had no influence on Zika virus neutralization (Fig 6C)—consistent with the data displayed in Fig 2 - and did not significantly affect Zika virus ADE (Fig 7A–7D).

### Role of broadly flavivirus cross-reactive antibodies in heterologous NT and ADE

**Fig 8:**
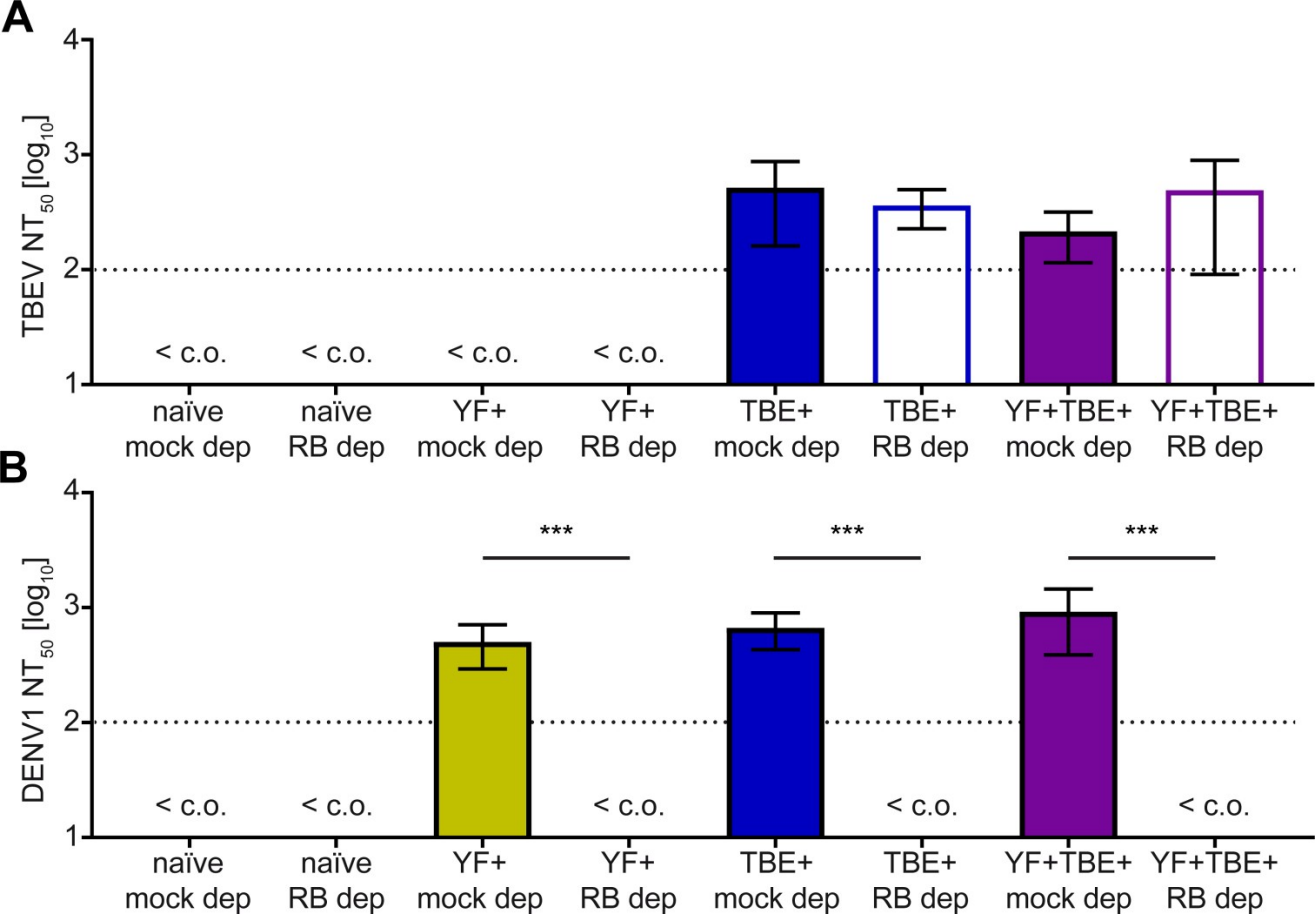
Effect of depletion of cross-reactive antibodies from serum pools with Rio Bravo virus E on TBE and dengue virus neutralization. (A) TBE virus NT. (B) Dengue virus NT. IgG-containing serum pools were further depleted with RB virus E protein as described in Methods. Colored columns: mock depletion, empty columns: RB depletion. The dotted line indicates the cut-off (c.o.) of the assay. Mean NT_50_ titers were calculated from two (A) or three (B) independent experiments. Error bars represent the range (A) or standard error of the mean (B). Significant differences between mock depletion and RB depletion are indicated by asterisks (2-way ANOVA with linear contrasts); ***, p < 0.001. TBEV, tick-borne encephalitis virus; DENV1, dengue virus serotype 1; mock dep, IgM and mock-depleted serum pool; RB dep, IgM and Rio Bravo virus E-depleted serum pool.

**Fig 9:**
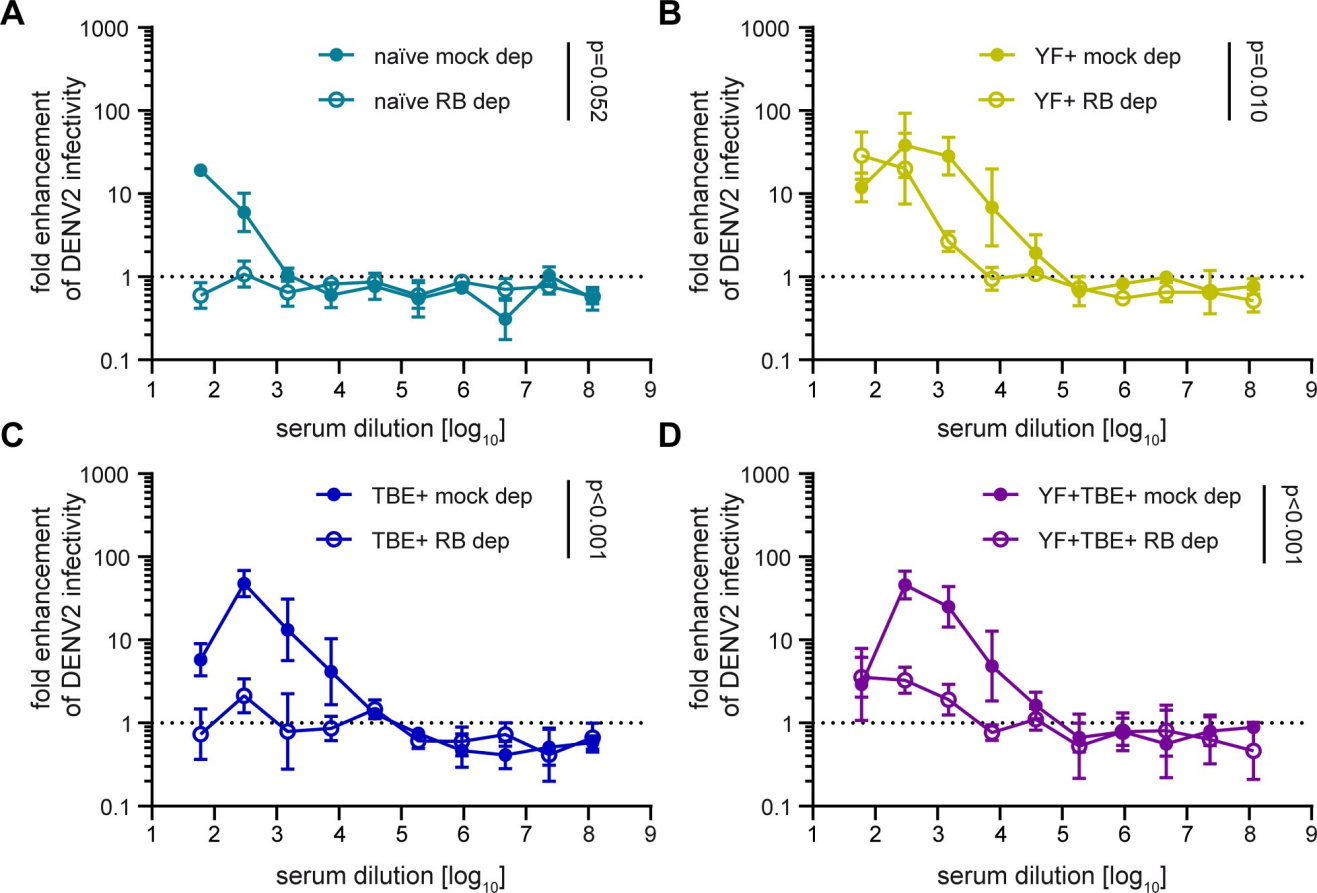
Effect of depletion of cross-reactive antibodies from serum pools with Rio Bravo E on ADE of dengue virus infection. Fcγ receptor-positive K562 cells were infected with dengue virus in the presence of serially diluted (A) flavivirus-naïve pool, (B) YF pre-vaccinated pool, (C) TBE pre-vaccinated pool, and (D) YF and TBE pre-vaccinated pool. IgG-containing serum pools were further depleted with RB E protein as described in Methods. Colored symbols: mock depletion, empty symbols: RB depletion. Data are shown as mean values +/- SEM from three independent experiments. Dotted line indicates no enhancement of infection. Comparison of fold-enhancement of dengue virus infection in the presence of depleted and mock-depleted pools was performed by applying a general linear model as described in Methods. p-values are indicated in the graphs. DENV2, dengue virus serotype 2; mock dep, IgM and mock-depleted serum pool; RB dep, IgM and Rio Bravo virus E-depleted serum pool.

There is evidence that the neutralizability by broadly cross-reactive antibodies can vary between different flaviviruses [19]. We therefore analyzed the four pools before and after depletion with the RB E protein in TBE and dengue virus NTs. As can be seen in Fig 8A, TBE virus was only neutralized by the pools from TBE-vaccinated individuals, and depletion of cross-reactive antibodies had no significant effect on this neutralizing activity. In contrast, dengue virus serotypes 1 and 2 were neutralized with similar titers (Fig 8B, S9 Fig) by all three pools from flavivirus pre-immune individuals, and this neutralizing activity was abolished by RB depletion (Fig 8B, S9 Fig). No cross-neutralization was observed with the naïve pool (Fig 8B, S9 Fig). These data suggest that the epitopes recognized by broadly cross-reactive antibodies are more accessible in infectious dengue than in TBE and Zika viruses (compare Fig 6C). This conclusion is also confirmed by the results of ADE assays with dengue virus serotype 2 (Fig 9), which showed that most of the enhancing activity could be removed by RB depletion with all four serum pools and therefore was due to cross-reactive antibodies.

### Distribution of antibody responses over time—single serum analyses

**Fig 10:**
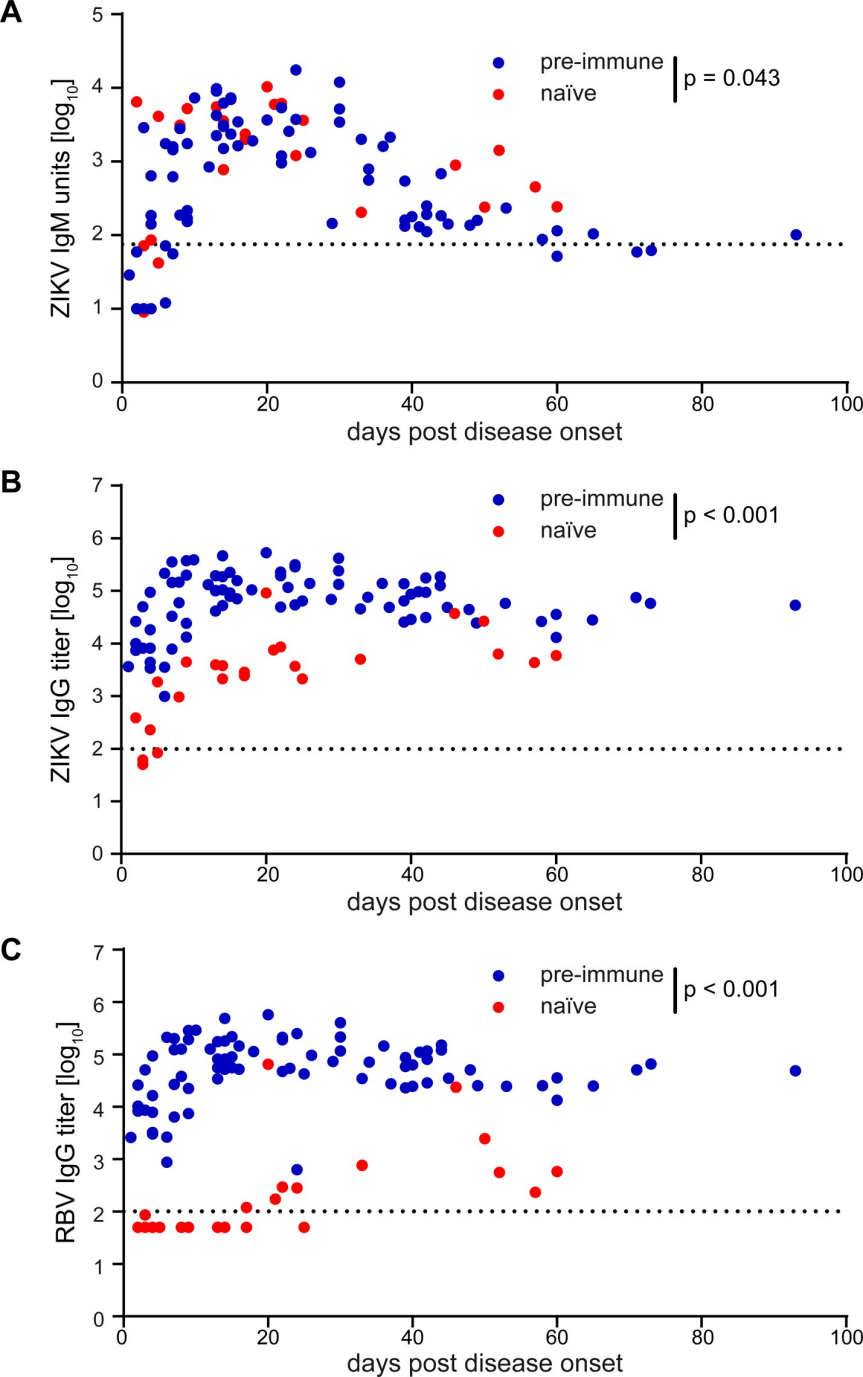
Quantification of Zika virus-specific IgM and IgG antibodies and broadly cross-reactive IgG antibodies using single serum samples from Zika cases. (A) Zika virus IgM ELISA units, (B) Zika virus IgG ELISA titers, and (C) Rio Bravo virus IgG ELISA titers of individual samples are plotted against days after disease onset. Data points represent means from two to three (A) or three (B, C) independent experiments. Flavivirus pre-immune patients are color-coded in blue, flavivirus naïve patients in red. Dotted lines indicate the cut-off of each assay. The significance of differences between the extents of naïve and pre-immune antibody responses were calculated based on the maxima reached, applying a general linear model as described in Methods. p-values are indicated in the graphs. ZIKV, Zika virus; RBV, Rio Bravo virus.

**Fig 11:**
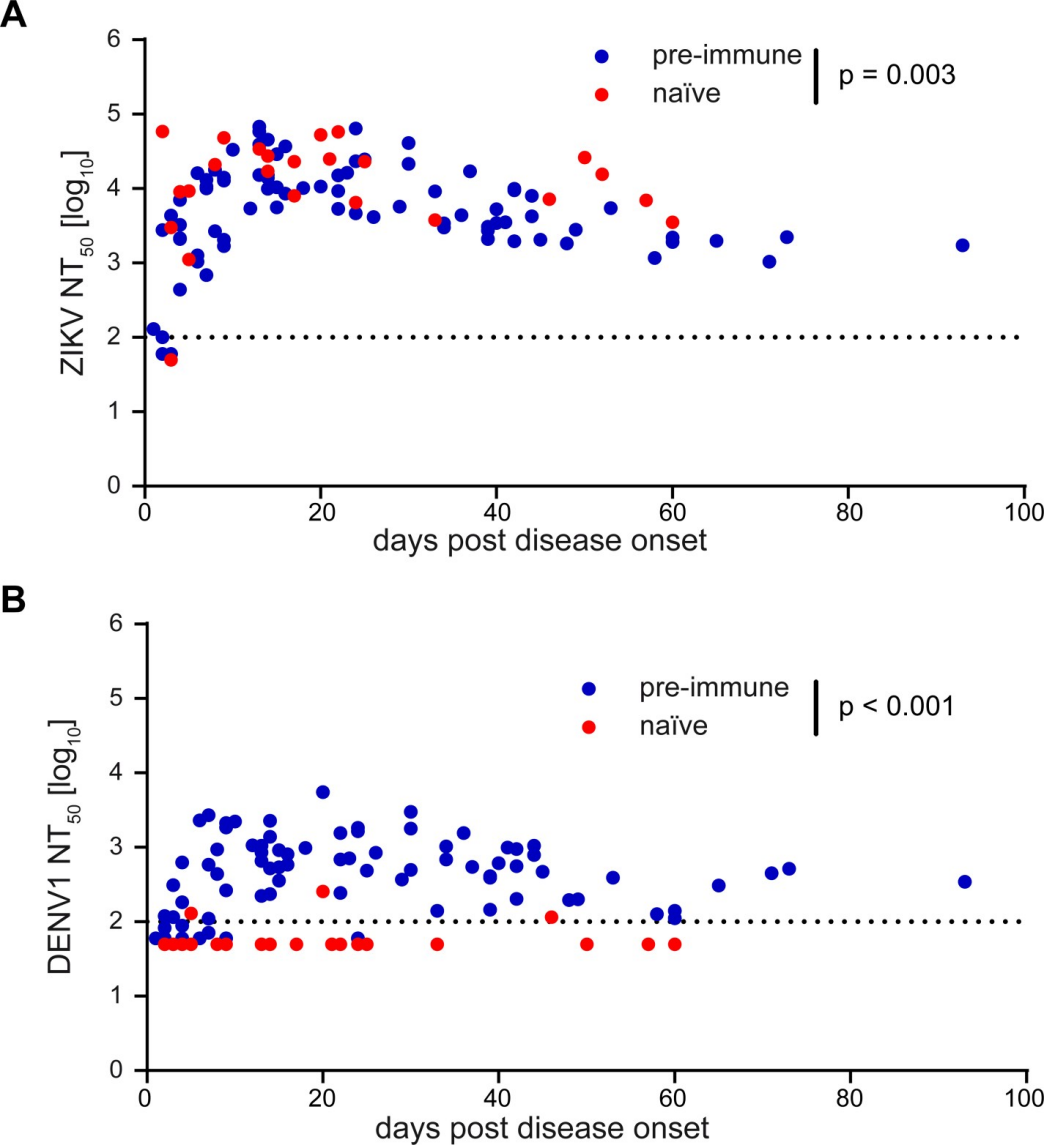
Zika and dengue virus neutralization tests using single serum samples from Zika cases. (A) Zika virus and (B) dengue virus NT_50_ titers from individual serum samples were plotted against days after disease onset. Data points represent mean NT_50_ titers from at least three independent experiments. Color code as in Fig 10. Dotted lines indicate the cut-off of each assay. The significance of differences between the extents of naïve and pre-immune antibody responses were calculated based on the maxima reached, applying a general linear model as described in Methods. p-values are indicated in the graphs. ZIKV, Zika virus; DENV1, dengue virus serotype 1.

The serum pools described above did not provide information about possible differences in the time course of antibody production in our cohorts. We therefore also compared individual serum samples from naïve and pre-immune Zika patients (Table 3), for which sufficient volumes were available, in Zika IgM and IgG ELISAs, RB IgG ELISA, as well as Zika and dengue NTs. For this comparison and based on the small number of Zika patients in the YF group, all samples from pre-vaccinated individuals were analyzed together. The samples span a time window of one to 93 days after disease onset, and the results are displayed in Figs 10 and 11.

The distribution of IgM antibodies appeared similar at first glance, with a rapid rise followed by a decline in both groups (Fig 10A). Statistical analysis, however, revealed significantly lower titers in the pre-immune group by a factor of 1.57 (Fig 10A), whereas no such difference was found with respect to the kinetics of IgM development (no significant difference between slopes of naïves and pre-immunes by applying a GLM as described in Methods, p = 0.187). The difference between the two groups was much more pronounced in IgG ELISAs with Zika and RB antigens (Fig 10B and 10C), with a faster and more extensive rise of IgG titers in the pre-immunes (significant difference between slopes of naïves and pre-immunes by applying a GLM as described in Methods, p < 0.001), consistent with an anamnestic booster response of broadly flavivirus cross-reactive antibodies.

The pattern of Zika virus NT appeared very similar with samples of the naïve and pre-immune groups (Fig 11A); reminiscent of what was observed in IgM ELISA (Fig 10A). However, statistical analysis revealed again significantly lower titers by a factor of 1.83 in the pre-immune group (Fig 11A). Consistent with the pool data (Fig 8B and S9 Fig), neutralization of dengue virus was observed with almost all samples from the pre-immune group, whereas most of the samples from the naïve group were negative (Fig 11B).

## Discussion

Pre-existing cross-reactive immunity can affect the antibody response upon infection with antigenically related viruses, both with respect to the kinetics and extent of the IgM response and to the specificities of populations of IgG antibodies induced. In our study, we analyzed the effect of prior TBE and/or YF vaccination on antibody responses to primary Zika virus infections and observed a strong anamnestic response in pre-vaccinated individuals, resulting in a high proportion of broadly cross-reactive IgG antibodies that accounted for 84 to 94% of the total E-response (Fig 6A). In naïve individuals, however, this proportion was much lower (Fig 6A). Since the magnitude of the newly formed Zika virus-specific IgG response was similar in naïve and pre-vaccinated groups (Fig 6A), the decisive effect of pre-immunity was a change in the proportion of IgG specificities that make up the antibody populations in post-infection sera, in favor of broadly cross-reactive antibodies. It can be assumed that these antibodies react with the FL epitope, since this site comprises the only patch of amino acid residues in E that is conserved across the viruses analyzed [19].

The FL appears to be cryptic in the cryo-EM (electron microscopy) structures of mature flaviviruses [19]. Consistent with this picture, broadly cross-reactive antibodies did not neutralize Zika and TBE viruses to a significant extent (Figs 6C and 8A). In contrast, the same antibodies were able to neutralize dengue virus (Fig 8B and S9 Fig), suggesting differences in the accessibility of broadly cross-reactive epitopes in different flaviviruses. The differential behavior observed can be related to stronger dynamic motions of the dengue virus envelope or incomplete maturation of viral particles, both of which can lead to a better exposure of the FL epitope [19, 21].

The different properties of infectious Zika and dengue viruses with respect to the exposure of broadly cross-reactive epitopes are not only reflected in NTs but also in analyses of ADE of infection of Fcγ receptor-expressing cells (Figs 7 and 9). Like neutralization, this phenomenon requires the binding of antibodies to infectious virus particles, albeit at sub-neutralizing concentrations [30]. Broadly cross-reactive antibodies boostered by Zika virus in the TBE and YF pre-vaccinated individuals only enhanced dengue but not Zika virus in our in vitro assays, consistent with a better accessibility of their epitopes in dengue viruses. Our data thus corroborate studies that have shown differences in conformational dynamics as well as maturation levels among different flaviviruses (and even strains of flaviviruses) [21], which have implications for virus neutralization and deserve further intensive research in the area of flavivirus vaccinology. It is therefore important to emphasize that the results obtained with dengue viruses (i.e. neutralization and strong ADE by cross-reactive antibodies, in contrast to Zika virus) may be biased by the specific properties of the virus strain used in the in vitro analyses. In particular, the results can be affected by a pronounced breathing behavior and partial immaturity, both of which result in a higher exposure of the conserved FL and thus may lead to an overestimation of the contribution of cross-reactive antibodies in these activities [19, 21]. Consistent with this notion, recent studies with dengue virus serotype 1 have shown that virus specimens taken directly from human plasma are predominantly mature (in contrast to cell culture grown virus) and are only poorly neutralized by antibodies recognizing the FL [65].

In our analyses we used a stable Zika virus E dimer (S4 Fig) which has a native antigenic structure and displays quaternary, E dimer-specific epitopes ([44], S4 Fig). Although more complex quaternary epitopes (generated by the herring-bone-like arrangement of E at the surface of mature viruses) have been identified by cryo EM analyses of Fab-virion complexes (reviewed in [18, 19, 66]) we show that corresponding antibodies in our polyclonal sera accounted for a maximum of ~ 10% of total Zika virus NT activity (S5 Fig).

Although Zika virus was not enhanced by broadly flavivirus cross-reactive antibodies in our study, infection enhancement of this virus was readily demonstrable with Zika virus-specific antibodies at sub-neutralizing concentrations, consistent with the generally accepted ADE mechanism [30]. An extremely important finding of our study is the fact that this infection enhancement can be potently suppressed by the presence of IgM antibodies (Figs 4 and 5), and this ability may be crucial in the early phase of flavivirus infections, when potentially enhancing antibodies already pre-exist or are strongly boostered. The in vivo relevance of an anti-ADE effect of IgM antibodies was recently shown in a mouse study with Zika virus, demonstrating that IgG-mediated ADE can be weakened by the presence of IgM [67]. Mechanistically, low-avidity Zika-virus specific IgG (as present early after infection, see also Fig 6B) might be sterically blocked by large pentameric IgM antibodies. IgM do not bind to Fcγ receptors [68], and can thus prevent virus complexes from binding to Fcγ receptors.

Analysis of virus neutralization before and after IgG depletion revealed that IgM accounted for approximately 30 to 50% of the total neutralizing activity in our samples of primary Zika virus infections, with a tendency of a lower contribution in the double-vaccinated group (Fig 3). These proportions are similar to those found in serum samples from primary dengue virus infections in an analysis of IgM- and IgG-mediated virus neutralization [69]. The virus neutralizing and anti-ADE activity of IgM (Figs 3, 4 and 5) highlight the general importance of IgM antibodies for controlling flavivirus clearance early after infection, also deduced from animal studies with West Nile virus and IgM-deficient mice [24].

It appears that the prominent role of IgM antibodies in virus neutralization in the early phase of human flavivirus infections and its function in protecting from severe forms of disease has been underappreciated so far. This is especially important in the context of sequential flavivirus infections in which IgM responses may be delayed and/or strongly diminished [14–16, 26, 27]. In the case of sequential dengue virus infections, IgM levels may even be reduced to non-detectable levels [14]. Animal studies suggest that this suppressive effect depends on the degree of antigenic relationship in such scenarios [16]. In the specific situations analyzed in our study, the impairment of Zika virus-specific IgM responses was not very strong but demonstrated to be significant (Fig 10A). It is apparent that the effects described are transient due to dynamic changes of the proportions and avidities of IgM and IgG antibodies in the early phase of infection. Whether and/or how the differences observed can affect durable immunity may be the subject of future studies performed with samples collected more than one year after infection.

Overall, our study highlights the potential effect of flavivirus vaccine-induced pre-immunity on the patterns of antibody responses in early stages of primary infection with a distantly related flavivirus, in this case Zika virus. The modulation observed, albeit with relatively small groups (especially the YF pre-vaccinated group) of adults, includes both the primary IgM response as well as the anamnestic booster of broadly cross-reactive IgG antibodies that are capable to mediate ADE of dengue virus infections in vitro. Considering the importance of IgM for protection early after infection and its ability to block IgG-mediated ADE, further studies on these phenomena in the context of different combinations of flavivirus infections and vaccinations appear to be warranted.

## Supporting information

S1 Fig Controls for the flavivirus IgG ELISA. (A) Titration curves of the positive control serum (described in Methods) and (B) results of 32 negative control sera (dilution 1:100) in ELISA with Zika, dengue serotype 2, RB and TBE virus E proteins. The cut-off used for titer calculations is shown as dotted line and corresponds to the mean absorbance value of the 32 negative samples plus 3 standard deviations.
(TIF)

S2 Fig Control of the Zika IgG avidity ELISA with early and late Zika virus post-infection serum samples. (A) Serum collected 3 weeks after disease onset and (B) after 6 months were tested with and without a step of urea exposure, as described in Methods.
(TIF)

S3 Fig Controls for the Zika IgM ELISA. (A) Standard curve of the positive control serum (described in Methods) and (B) results of 32 negative control sera in ELISA with Zika virus E protein. The cut-off is shown as dotted line and corresponds to the mean arbitrary units of the 32 negative samples plus 4 standard deviations.
(TIF)

S4 Fig Analysis of the recombinant Zika virus E protein used in this study. (A) Rate zonal ultracentrifugation of untreated Zika virus E protein (red solid line), and after SDS-treatment (red dotted line). The monomeric WN virus E protein (blue solid line) served as a control. M and D indicate positions of monomers and dimers, respectively. (B) Size-exclusion chromatograms of Zika virus E (upper panel) and the monomeric WN virus E (lower panel). (C) ELISA with Zika virus E protein and EDE-specific monoclonal antibodies (C8 and C10), reacting with E dimers but not monomers [44].
(TIF)

S5 Fig Results of Zika virus neutralization of samples before and after antibody depletion with the Zika virus E dimer. (A) Serum pools. (B) Individual serum samples. The % residual neutralization after depletion is indicated in the empty columns. Mean titers were calculated from two independent experiments and error bars represent the range. Colored columns: mock depletion (mock dep), empty columns: Zika virus E depletion (ZIKV dep). n.t., not tested, because of volume limitation of the YF+ pool. S1 to S4: individual sera from each of the 4 groups.
(TIF)

S6 Fig Zika virus IgG and IgM ELISA after IgG depletion of serum pools. Serum pools were depleted with protein G columns as described in Methods. (A) Zika virus IgG ELISA showing more than 99% depletion of IgG. (B) Zika virus IgM ELISA to control for loss of IgM antibodies during this procedure. Colored columns: mock depletion, empty columns: IgG depletion. The dotted line indicates the cut-off (c.o.) of the assay. Mean values were calculated from two independent experiments and error bars represent the range.
(TIF)

S7 Fig Zika virus IgM and IgG ELISA after IgM depletion of serum pools. Serum pools were depleted with anti-IgM agarose beads as described in Methods. (A) Zika virus IgM ELISA showing more than 99% depletion of IgM. (B) Zika virus IgG ELISA to control for loss of IgG antibodies during this procedure. Colored columns: mock depletion, empty columns: IgM depletion. The dotted line indicates the cut-off (c.o.) of the assay. Mean values were calculated from two independent experiments and error bars represent the range.
(TIF)

S8 Fig Rio Bravo IgG ELISA after depletion of cross-reactive antibodies with Rio Bravo virus E protein. Serum pools were depleted with RB virus E protein as described in Methods. Colored columns: mock depletion, empty columns: RB depletion. The dotted line indicates the cut-off (c.o.) of the assay. Mean values were calculated from two independent experiments and error bars represent the range.
(TIF)

S9 Fig Results of neutralization of dengue 1 and 2 viruses by serum pools without and with depletion of cross-reactive antibodies with Rio Bravo virus E protein. The dotted line indicates the cut-off (c.o.) of the assay. Mean values were calculated from two independent experiments and error bars represent the range. mock dep, mock-depleted serum pool; RB dep, Rio Bravo virus E-depleted serum pool.
(TIF)

S1 Table Statistical comparisons of pool and single serum data of the analyses displayed in Fig 2A, 2B, 2C, 2D and 2F. (DOCX)
