# Supplementary figures and images for: Impact of flavivirus vaccine-induced immunity on primary Zika virus antibody response in humans
Source: PLoS Negl Trop Dis. 2020 Feb 4;14(2):e0008034. doi: 10.1371/journal.pntd.0008034 (PMC7021315; doi:10.1371/journal.pntd.0008034)

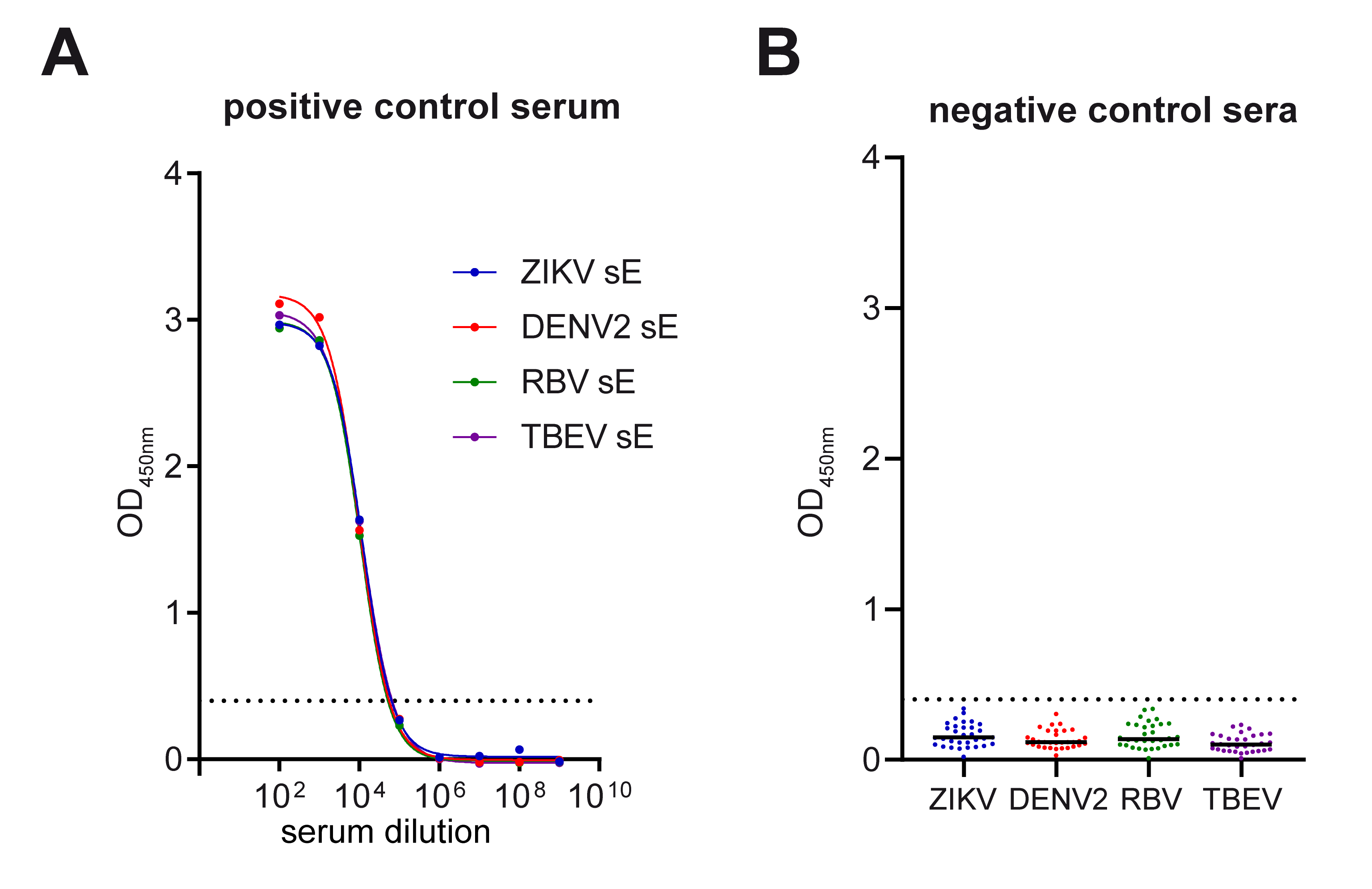

Supplement: S1 Fig — (A) Titration curves of the positive control serum (described in Methods) and (B) results of 32 negative control sera (dilution 1:100) in ELISA with Zika, dengue serotype 2, RB and TBE virus E proteins. The cut-off used for titer calculations is shown as dotted line and corresponds to the mean absorbance value of the 32 negative samples plus 3 standard deviations. (TIF) [file pntd.0008034.s001.tif]

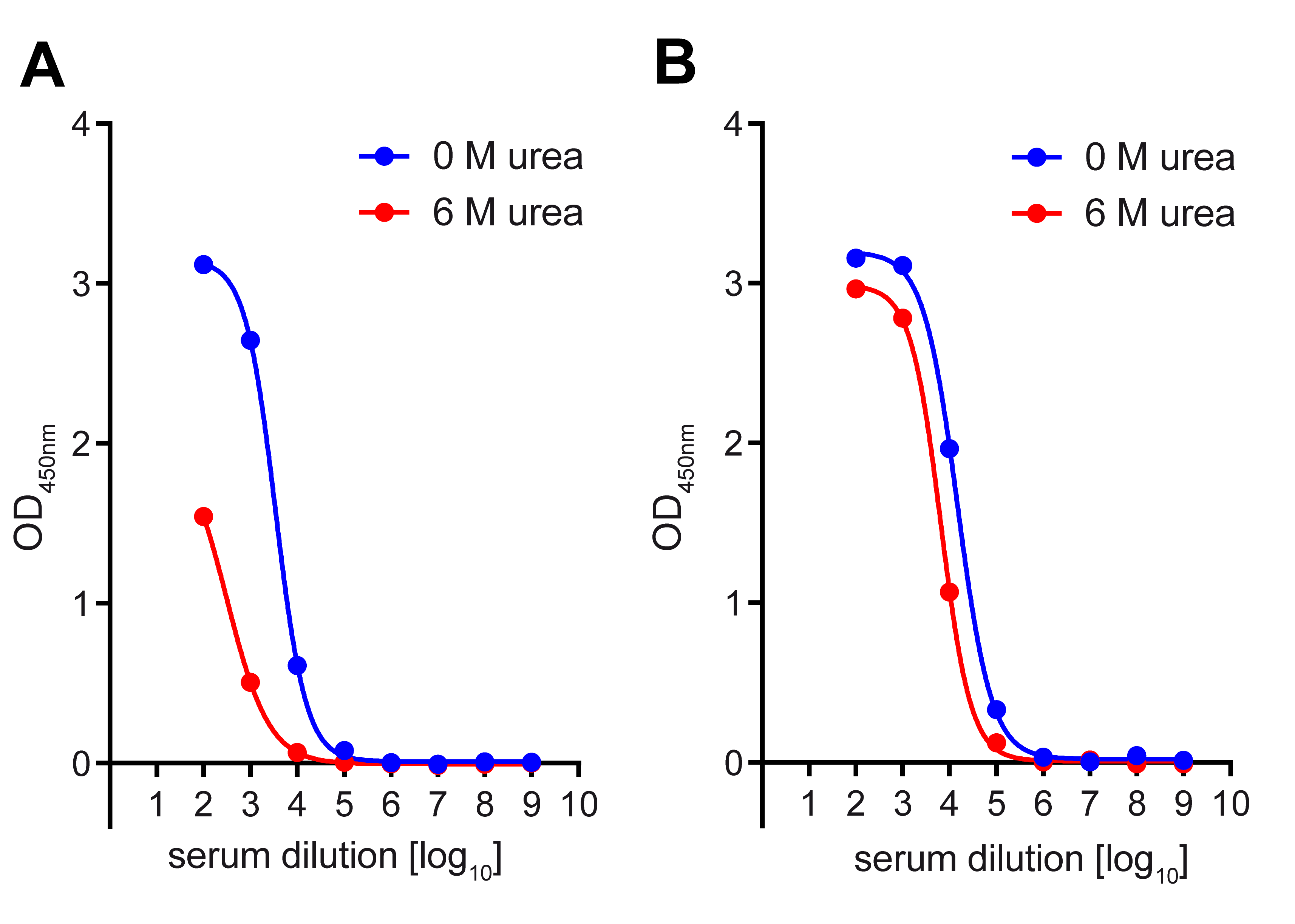

Supplement: S2 Fig — (A) Serum collected 3 weeks after disease onset and (B) after 6 months were tested with and without a step of urea exposure, as described in Methods. (TIF) [file pntd.0008034.s002.tif]

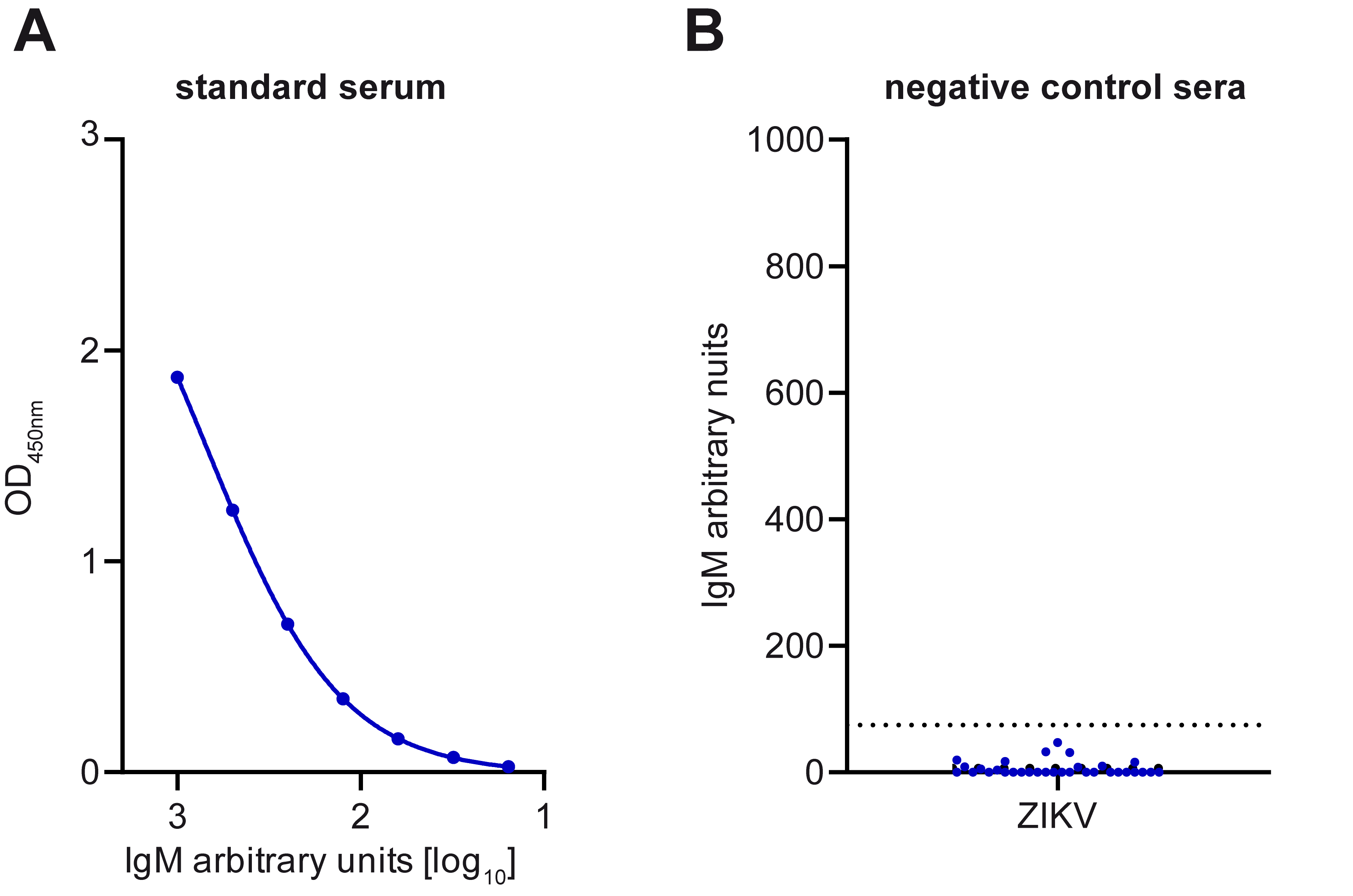

Supplement: S3 Fig — (A) Standard curve of the positive control serum (described in Methods) and (B) results of 32 negative control sera in ELISA with Zika virus E protein. The cut-off is shown as dotted line and corresponds to the mean arbitrary units of the 32 negative samples plus 4 standard deviations. (TIF) [file pntd.0008034.s003.tif]

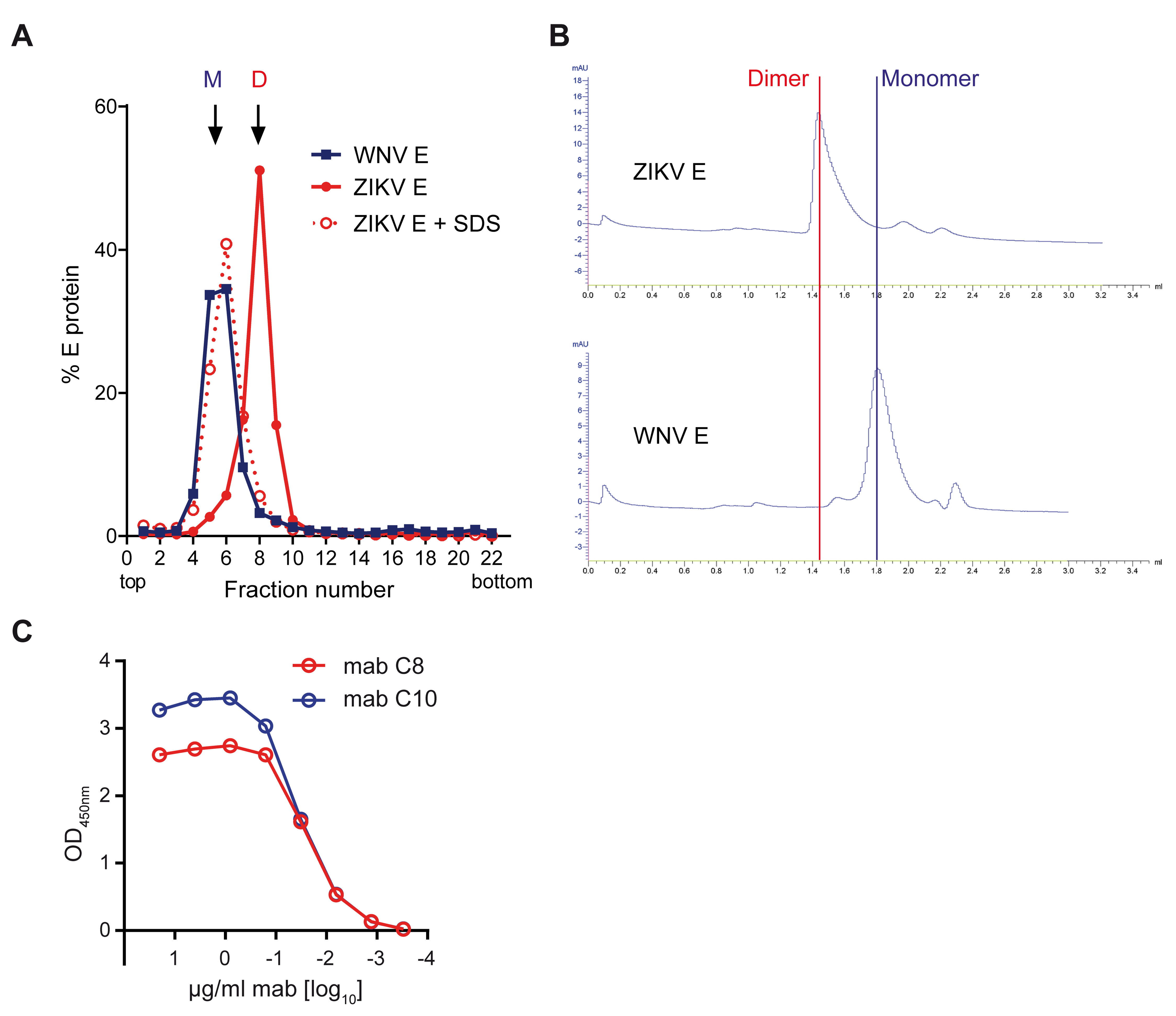

Supplement: S4 Fig — (A) Rate zonal ultracentrifugation of untreated Zika virus E protein (red solid line), and after SDS-treatment (red dotted line). The monomeric WN virus E protein (blue solid line) served as a control. M and D indicate positions of monomers and dimers, respectively. (B) Size-exclusion chromatograms of Zika virus E (upper panel) and the monomeric WN virus E (lower panel). (C) ELISA with Zika virus E protein and EDE-specific monoclonal antibodies (C8 and C10), reacting with E dimers but not monomers [44]. (TIF) [file pntd.0008034.s004.tif]

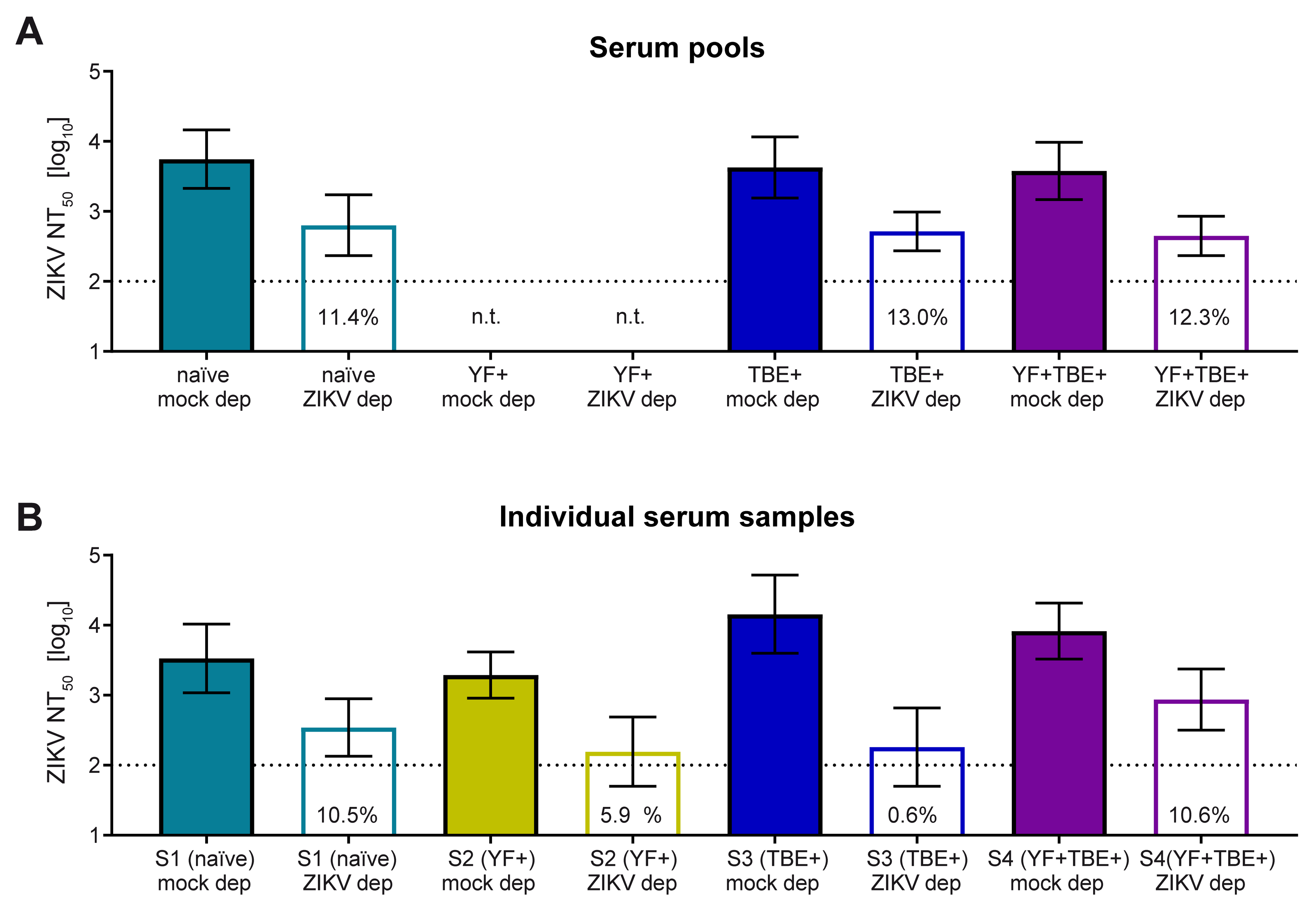

Supplement: S5 Fig — (A) Serum pools. (B) Individual serum samples. The % residual neutralization after depletion is indicated in the empty columns. Mean titers were calculated from two independent experiments and error bars represent the range. Colored columns: mock depletion (mock dep), empty columns: Zika virus E depletion (ZIKV dep). n.t., not tested, because of volume limitation of the YF+ pool. S1 to S4: individual sera from each of the 4 groups. (TIF) [file pntd.0008034.s005.tif]

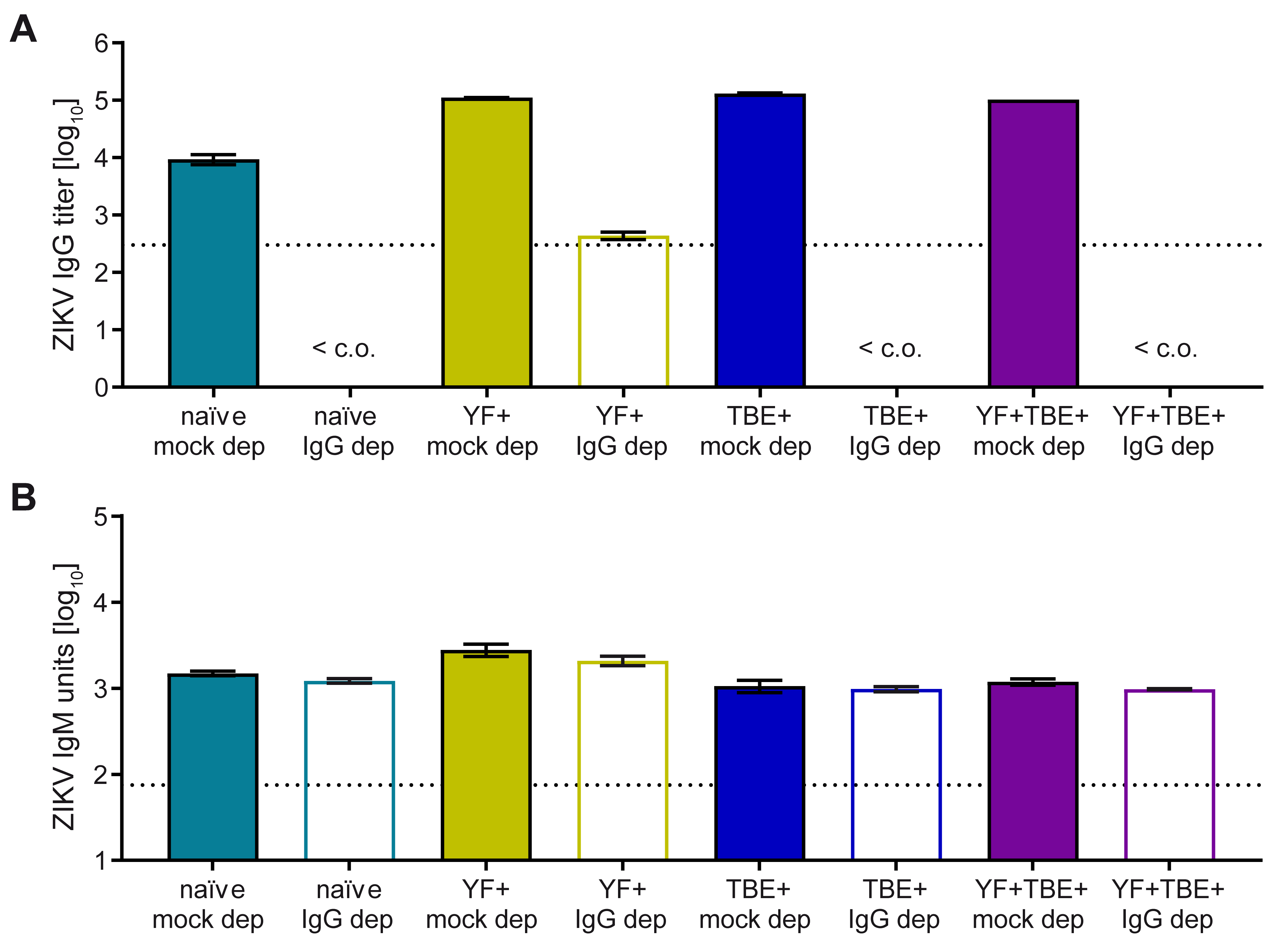

Supplement: S6 Fig — Serum pools were depleted with protein G columns as described in Methods. (A) Zika virus IgG ELISA showing more than 99% depletion of IgG. (B) Zika virus IgM ELISA to control for loss of IgM antibodies during this procedure. Colored columns: mock depletion, empty columns: IgG depletion. The dotted line indicates the cut-off (c.o.) of the assay. Mean values were calculated from two independent experiments and error bars represent the range. (TIF) [file pntd.0008034.s006.tif]

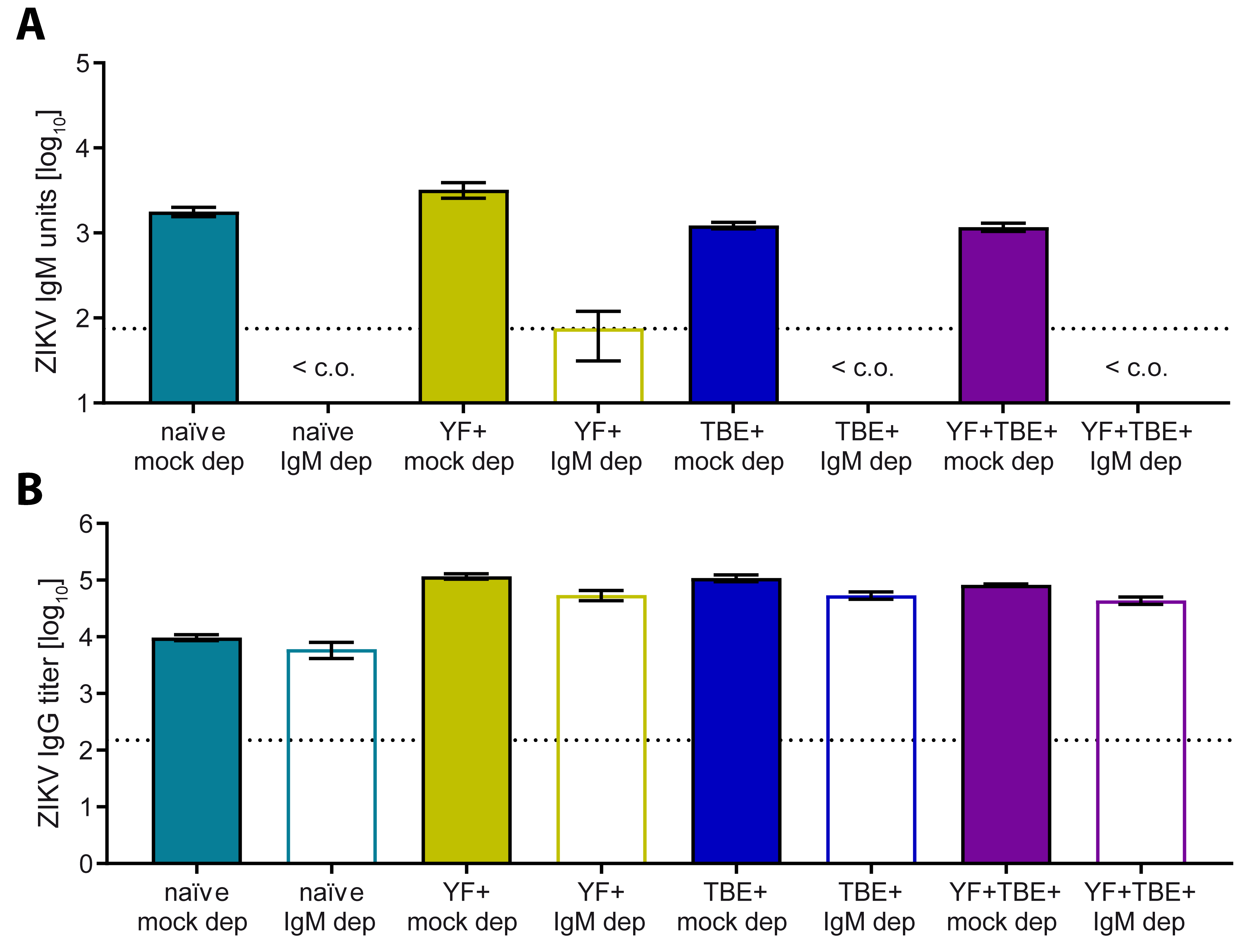

Supplement: S7 Fig — Serum pools were depleted with anti-IgM agarose beads as described in Methods. (A) Zika virus IgM ELISA showing more than 99% depletion of IgM. (B) Zika virus IgG ELISA to control for loss of IgG antibodies during this procedure. Colored columns: mock depletion, empty columns: IgM depletion. The dotted line indicates the cut-off (c.o.) of the assay. Mean values were calculated from two independent experiments and error bars represent the range. (TIF) [file pntd.0008034.s007.tif]

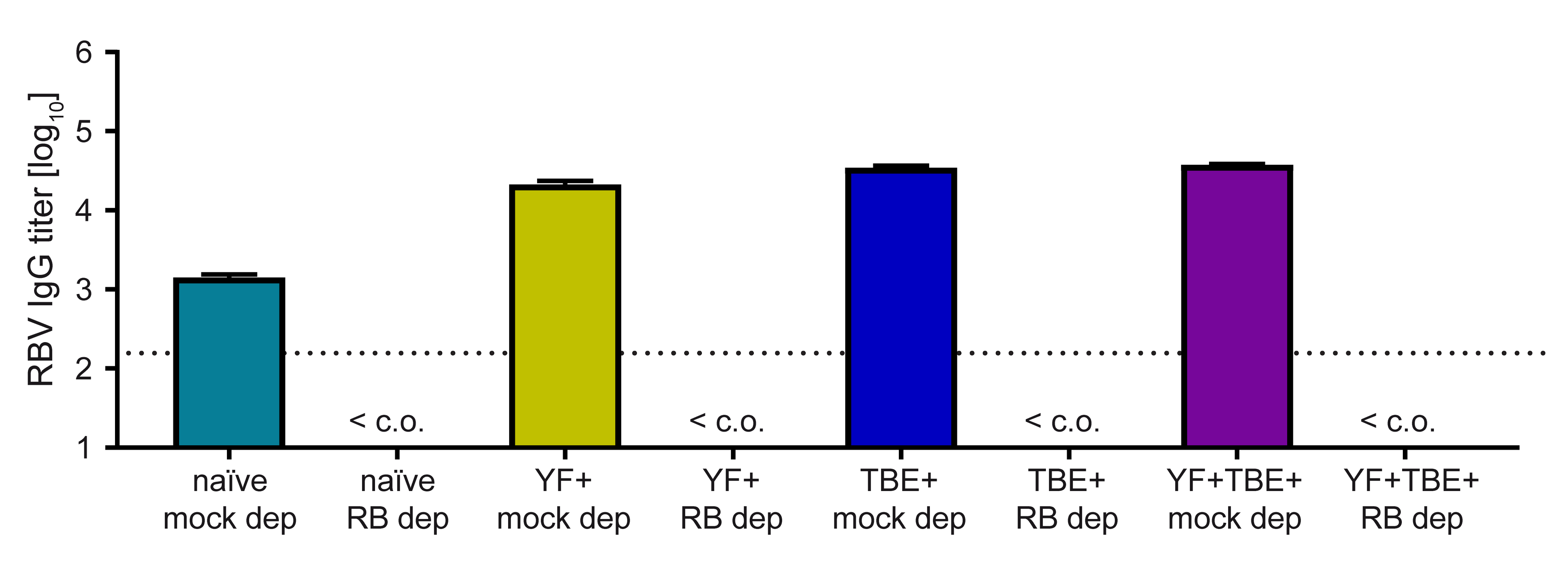

Supplement: S8 Fig — Serum pools were depleted with RB virus E protein as described in Methods. Colored columns: mock depletion, empty columns: RB depletion. The dotted line indicates the cut-off (c.o.) of the assay. Mean values were calculated from two independent experiments and error bars represent the range. (TIF) [file pntd.0008034.s008.tif]

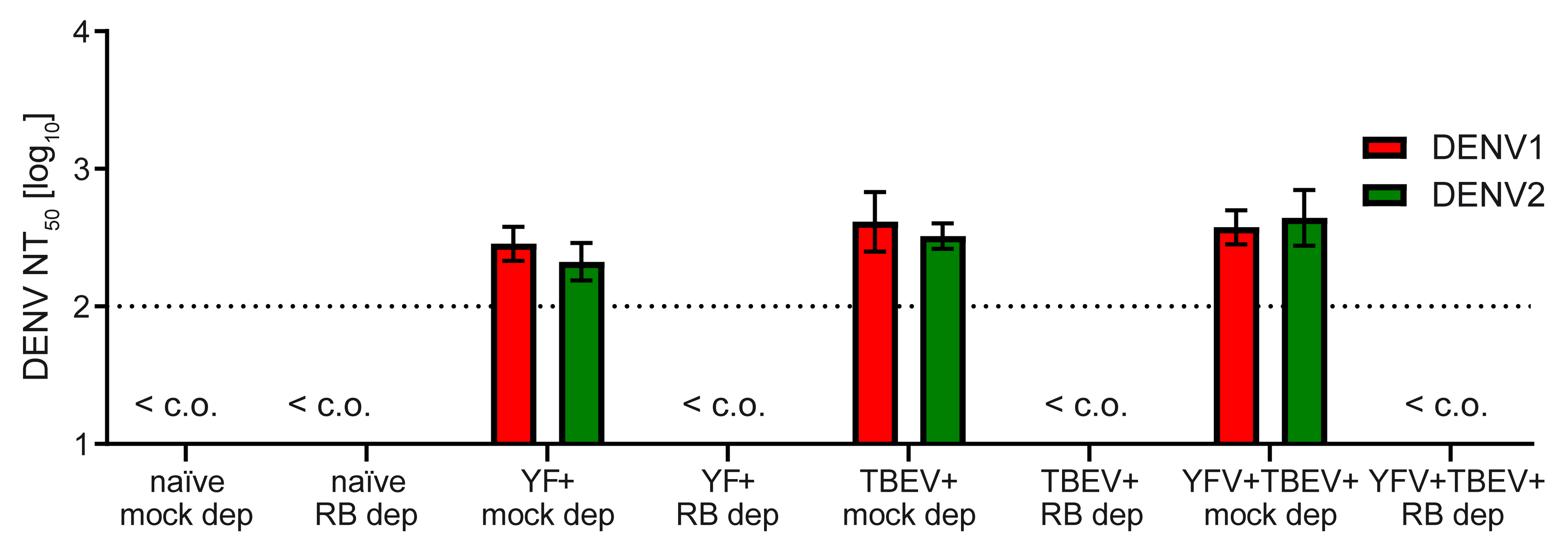

Supplement: S9 Fig — The dotted line indicates the cut-off (c.o.) of the assay. Mean values were calculated from two independent experiments and error bars represent the range. mock dep, mock-depleted serum pool; RB dep, Rio Bravo virus E-depleted serum pool. (TIF) [file pntd.0008034.s009.tif]
