# Supplementary material for: Impact of flavivirus vaccine-induced immunity on primary Zika virus antibody response in humans
Source: PLoS Negl Trop Dis. 2020 Feb 4;14(2):e0008034. doi: 10.1371/journal.pntd.0008034 (PMC7021315; doi:10.1371/journal.pntd.0008034)
Supplement: S1 Table — (DOCX) [file pntd.0008034.s010.docx]

S1 Table: Statistical comparisons of pool and single serum data of the analyses displayed in Fig 2A, B, C, D, and F.

|  |  | **naive** | | **YF+** | | **TBE+** | | **YF+TBE+** | |
| --- | --- | --- | --- | --- | --- | --- | --- | --- | --- |
|  |  | **pool** | **single sera** | **pool** | **single sera** | **pool** | **single sera** | **pool** | **single sera** |
| ZIKV IgM units | **mean**  (SEM) | **1779**  (227) | **2030**^a^  (845) | **3230**  (661) | **4432**  (2603) | **1224**  (107) | **1679**^a^  (638) | **1172**  (131) | **896**^a^  (612) |
| Fig 2A | **^b^p=** | **0.384** | | **0.611** | | **0.390** | | **0.150** | |
| RBV IgM absorbance | **mean**  (SEM) | **0.02**  (0.01) | **0.02**  (0.01) | **0.09**  (0.01) | **0.13**  (0.09) | **0.02**  (0.00) | **0.02**  (0.01) | **0.03**  (0.00) | **0.05**^a^  (0.02) |
| Fig 2B | **^b^p=** | **0.988** | | **0.297** | | **0.788** | | **0.921** | |
| ZIKV IgG titers | **mean**  (SEM) | **11252**  (1092) | **10430**  (3720) | **121817**  (3822) | **155793**  (89831) | **131266**  (4776) | **118954**  (31308) | **116617**  (7609) | **106101**  (21710) |
| Fig 2C | **^b^p=** | **0.187** | | **0.530** | | **0.209** | | **0.504** | |
| RBV IgG titers | **mean**  (SEM) | **3633**  (417) | **2857**  (2319) | **98050**  (5988) | **142450**  (88960**)** | **87920**  (4386) | **94519**  (27484) | **82670**  (4083) | **87959**  (19008**)** |
| Fig 2D | **^b^p=** | **0.002** | | **0.736** | | **0.636** | | **0.872** | |
| ZIKV NT_50_ titers | **mean**  (SEM) | **4985**  (1199) | **9793**  (2986) | **6160**  (1815) | **7636**  (2937) | **3461**  (839) | **6120**  (1708) | **3215**  (737) | **6021**  (1883) |
| Fig 2F | **^b^p=** | **0.350** | | **0.869** | | **0.635** | | **0.409** | |

^a^ in figure 2A, one single serum from the naïve, TBE+ and YF+TBE and in figure 2B, one serum from YF+TBE+ pool was not tested due to volume limitations.

^b^ Statistical comparisons of pool and single serum data were performed by applying a general linear model (GLM) with the log link function.
